# Voltage-Driven Generation of Ferromagnetism in a Magneto-Ionically Active Antiferromagnet Enabling Room-Temperature Exchange Bias

**DOI:** 10.1021/acsnano.5c19864

**Published:** 2026-03-23

**Authors:** Simone Privitera, Zheng Ma, Hugo Gómez-Torres, Aitor Arredondo-López, Maciej Oskar Liedke, Eric Hirschmann, Andreas Wagner, Huan Tan, Pau Solsona, Alberto Quintana, Thiago Dias, Diane Gouéré, Elmer Monteblanco, Dafiné Ravelosona, Nuria Del-Valle, Carles Navau, Aitor Lopeandia, Jordi Sort, Enric Menéndez

**Affiliations:** † Departament de Física, Universitat Autònoma de Barcelona, 08193 Cerdanyola del Vallès, Spain; ‡ Catalan Institute of Nanoscience and Nanotechnology (ICN2), CSIC and BIST, Campus UAB, 08193 Cerdanyola del Vallès, Spain; § Institute of Radiation Physics, 28414 Helmholtz-Zentrum Dresden–Rossendorf, Dresden 01328, Germany; ∥ 730335 Universidade Tecnológica Federal do Paraná, Campus Dois Vizinhos, Estrada para Boa Esperança, km 04, 85660-000 Dois Vizinhos PR, Brazil; ⊥ Spin-Ion Technologies, 10 Boulevard Thomas Gobert, Palaiseau 91120, France; # Centre de Nanosciences et de Nanotechnologies, CNRS, Université Paris-Saclay, 10 Boulevard Thomas Gobert, Palaiseau 91120, France; ¶ Institució Catalana de Recerca i Estudis Avançats (ICREA), Pg. Lluís Companys 23, 08010 Barcelona, Spain

**Keywords:** voltage control of magnetism, magneto-ionics, antiferromagnetism, exchange bias, nanocalorimetry

## Abstract

Magneto-ionics–as the voltage-driven control of magnetic properties through ionic motion and redox processes–offers a promising route toward energy-efficient spintronic devices. Exchange bias, being the unidirectional anisotropy arising from interfacial coupling between antiferromagnets and ferromagnets, plays a central role in spintronics. Here, we demonstrate reversible, room-temperature magneto-ionic generation, suppression, and modulation of exchange bias within a 50 nm-thick antiferromagnetic, magneto-ionically active NiCoO layer. Instead of relying on field cooling to set exchange bias, an applied magnetic field during the growth promotes alignment of the antiferromagnetic spin sublattices, producing a preferential unidirectional orientation. Gating drives oxygen-ion migration along columnar grain boundaries, partially reducing NiCoO and forming ferromagnetic NiCo clusters that couple to the antiferromagnetic matrix. The exchange bias can be controlled by tuning the Ni/Co ratio, which adjusts the Néel temperature, and by varying the actuation time and voltage amplitude which control ferromagnetic cluster size. Micromagnetic simulations reveal that the exchange bias originates from the interfacial uncompensated spins exhibiting partial ferromagnetic-like behavior. This single-layer approach, together with the voltage-controlled formation and tuning of exchange bias without heat treatments, simplifies fabrication and offers a framework for low-power antiferromagnetic spintronic devices.

## Graphical abstract

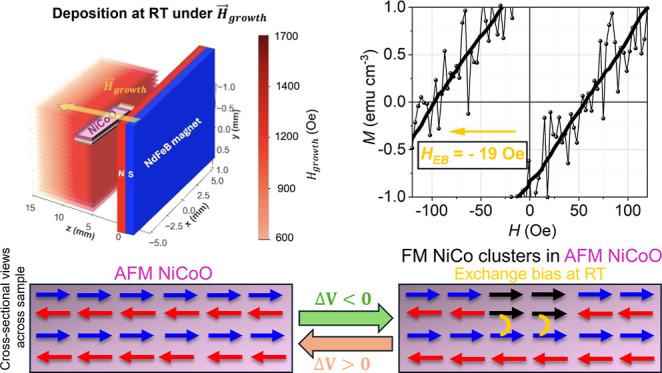

## Introduction

Magneto-ionics, which exploits voltage-driven ion transport and redox reactions to manipulate magnetism in a nonvolatile manner, has emerged as a compelling route for revolutionizing spin-electronics from an energy-efficiency viewpoint and for the development of novel data-processing paradigms, such as neuromorphic computing.^1,2^ Even though magneto-ionics can involve various types of ions, such as O^2–^,^3–7^ N^3–^,^8,9^ H^+^,^10^ Li^+^,^11^ F^–^ ^12^ or OH^–^,^13^ oxygen stands out due to its chemical stability, number of materials available, and compatibility with complementary metal-oxide semiconductor (CMOS) technology. Magneto-ionics has been widely applied to control the properties of ferromagnetic (FM) materials (e.g., coercivity,^7^ saturation magnetization,^3,8,14^ magnetic anisotropy,^5,7^ Curie temperature^15,16^ or the Dzyaloshinskii–Moriya interaction,^17^ among others), eventually enabling transformations between paramagnetic and ferromagnetic phases^3,8^ and transitions between vortex and single domain states.^18^ Magneto-ionic can also tailor the RKKY interaction^19^ in synthetic antiferromagnets, as well as exchange bias (EB) in ferromagnetic-antiferromagnetic (AFM) heterostructures.^20–36^ The latter is particularly relevant for the voltage control of spin valves and magnetic tunnel junctions, which constitute the fundamental building blocks of spintronic technologies. To date, several magneto-ionic approaches have been employed to control EB via liquid or solid-state gating. However, fully voltage-programmable exchange bias, encompassing its generation, suppression, and continuous modulation at room temperature (RT), has yet to be realized. Existing demonstrations often rely on complex heterostructures that hinder scalability and require thermal treatments that compromise energy efficiency. Achieving reliable EB control through magneto-ionics remains crucial, as antiferromagnets offer the stability and scalability required for high-density integration, and could enable energy-efficient, voltage-programmable AFM spintronic devices.^33–35^ Liu et al. demonstrated upon field cooling, the modulation of EB in a Gd/NiCoO/CoNi system, where initial CoNi formation is driven by chemical reactivity differences (not by voltage means), and additional CoNi originates from the voltage-driven reduction of NiCoO.^21–23^ In perovskite oxide heterostructures, such as La_0.8_Sr_0.2_CoO_3_/La_0.67_Sr_0.33_MnO_3_, oxygen migration can generate EB but at very low temperatures (5 K).^24^ Similarly, multilayer stacks like Pt/[Co/Ni]/HfO_2_ require long voltage pulses and low temperatures (<200 K) to sustain EB.^25,26^ Beyond oxygen, other mobile ions have been explored for EB control, offering faster response time and lower threshold voltage but still requiring high temperature annealing and sometimes showing limited stability. H^+^-based systems can modulate EB in milliseconds as demonstrated by Beach et al.^27,28^ in NiO/Pd/Co/Pd/GdO_*x*_/Au and GdO_*x*_/Co_0.8_Ni_0.2_O/Co stacks via gating-induced transport of hydrogen, sourced by water dissociation in high humidity atmospheres. N^3–^ diffusion in Ta/MnN/CoFe/Ta stacks^29^ and Li^+^ intercalation in a lithium-ion battery structure LiCoO_2_/Lithium-Ion Conducting Glass (LICGC)/NiO/Co^31^ can also produce EB, typically involving high temperature processing. Remarkably, most previous studies have achieved magneto-ionic control of exchange bias in FM/AFM bilayers, where the applied voltage modifies either the FM or the AFM phase through electric-field-induced ion motion. In all cases, field cooling was required to establish exchange bias, a process that undermines energy efficiency. Developing approaches that enable direct, voltage-driven modulation of exchange bias–without field cooling and preferably within a single magneto-ionic layer–would constitute a relevant step toward AFM spintronics. While magneto-ionics has been widely applied to ferromagnetic systems, its potential in tuning the properties of antiferromagnetic materials remains overlooked. Here, we report on the voltage-programmable control (i.e., generation, suppression, and modulation) at RT of EB in a single NiCoO film, without any prethermal activation or field cooling. Domain-oriented AFM NiCoO films with tunable Ni/Co ratios (i.e., Ni_*x*_Co_1‑*x*_O)–and hence tailored Néel temperatures (*T*_*N*_)–are grown at RT by reactive sputtering under an applied magnetic field. NiCoO is intentionally selected as a model system in which antiferromagnetic order and ionic transport can be balanced. While Ni incorporation strengthens antiferromagnetic coupling and supports room-temperature exchange bias,^20^ excessive Ni content tends to suppress ion mobility.^32^ The Ni concentration is therefore minimized to maintain antiferromagnetic order while ensuring the voltage-driven formation of ferromagnetic clusters at room temperature. During magneto-ionic actuation–measured along the direction of the growth field–voltage-driven reduction of NiCoO leads to the formation of NiCo clusters along columnar grain boundaries, which act as diffusion channels. These generated clusters create AFM/FM coupled regions embedded within the AFM matrix. Our approach offers several key advantages over prior work. First, it enables fully voltage-driven, room-temperature ON/OFF control of EB, rather than mere voltage modulation. Second, the process is inherently simple, relying on a single-layer architecture without the need for field cooling. Overall, this work demonstrates a scalable, low-power platform where the antiferromagnet itself serves as an active magneto-ionic component, in contrast to conventional EB devices where the AFM usually plays a passive role and need others layers to be useful. These features provide a basis for next-generation energy-efficient AFM spintronic devices.^34^

## Results and Discussion

50 nm-thick AFM polycrystalline Ni_*x*_Co_1‑*x*_O thin films (with *x* = 0, 0.2, 0.25, 0.35, and 0.4) were grown at RT using DC reactive cosputtering, aiming to systematically tune the *T*_N_ through the controlled adjustment of the Ni/Co ratio. The films were grown on partly masked Si(100) substrates, previously coated with 20 nm of Ti and 60 nm of Cu, which serve as bottom electrode (Figure 1a,b). The Ni_*x*_Co_1‑*x*_O film tends to show a columnar-like microstructure (Figure 1b), as often seen in Co oxide systems.^3^ During deposition, an out-of-plane magnetized NdFeB permanent magnet with a saturation magnetization of 1145.2 emu·cm^–3^ was positioned adjacent to the sample (Figure 1a), such that its stray magnetic field (*H*_growth_) promotes the preferential orientation of AFM domains during film growth. This results in an oriented AFM state that defines the anisotropy direction of the AFM matrix without the need for postdeposition field cooling. This behavior is favored by the A-type AFM structure of NiCoO, in which spins are ferromagnetically coupled within atomic planes while adjacent planes are coupled antiferromagnetically (Figure 1c). All films were grown under *H*_growth_ unless stated otherwise. Figure 1a also shows the sample position during deposition, together with the spatial distribution, uniformity, and magnitude of the magnetic field generated by the permanent magnet (see Methods). The X-ray diffraction (XRD) pattern of CoO, which serves as a reference, confirms the formation of a rocksalt-type structure (see Figure S1, Supporting Information), which is linked to the existence of AFM behavior. The peak located at 2θ ≈ 61.5° is unambiguously associated with CoO (not overlapping with Co_3_O_4_), which corresponds to the (220) reflection of the rocksalt phase of CoO (PDF 00-001-1227). In Ni_0.25_Co_0.75_O, the (220) peak is shifted to a slightly higher 2θ angle compared to CoO, falling between the (220) reflections of pure CoO and NiO, evidencing the formation of a ternary rocksalt NiCoO phase.^22^ To investigate the role of *H*_growth_ in film microstructure, CoO films, rather than NiCoO, were grown with and without the permanent magnet. This choice minimizes variables, isolates the effect of the magnetic field, and ensures a high signal-to-noise ratio, while excluding any additional influence from Ni. The films were characterized by θ/2θ XRD. As shown in Figure S1c, the (220) XRD peak of the film grown in the presence of the permanent magnet exhibits a slightly narrower full width at half-maximum (fwhm), consistent with a larger crystallite size. Rietveld refinements of the full θ/2θ diffraction patterns were performed using the MAUD software to quantify the crystallite size. While the film grown without an applied magnetic field shows a crystallite size of 37 ± 3 nm, the film grown under a magnetic field exhibits a larger crystallite size of 44 nm ± 3 nm. These results indicate that the presence of a magnetic field during growth has a subtle effect on the film microstructure, tending to promote slightly larger crystallites. Nanocalorimetry was used to determine the *T*_N_ of the Ni_*x*_Co_1‑*x*_O films grown under *H*_growth_ (Figure 1d). The peak of the heat capacity at a constant pressure (*C*_P_) vs. temperature *T* shifts to higher temperatures as the Ni content increases, reflecting the corresponding rise in *T*_N_ (see Methods). Moreover nanocalorimetry measurements of a NiCoO film grown with and without *H*_growth_ reveal distinct magnitudes of heat capacity near the AFM-paramagnetic transition, identified by the peak position *T*_N_: the film grown under *H*_growth_ exhibits an enhanced heat capacity (Figure 1e). Additionally, the inset of Figure 1e reveals the accumulated entropy variation (Δ*S*) associated with the order–disorder transition in the 240–370 K range, where experimental data is available. For comparable sample masses, the specimen grown under a magnetic field exhibits an entropy change about 30% larger than that of the sample grown without the field. This enhanced Δ*S* indicates a greater volume fraction involved in the AFM transition, consistent with an increased domain size. Atoms located at domain boundaries or in the interdomain regions are more weakly coupled and thus do not contribute appreciably to the transition or to the entropy change.^37^ The reduced entropy variation in the sample grown without field is therefore attributed to a higher fraction of such loosely coupled atoms, likely associated with an enhanced AFM domain density. This behavior is consistent with a more ordered AFM state produced by the field-assisted growth: *H*_growth_ aligns AFM domains during deposition, creating a preferential direction of domain orientation.

**Figure 1:**
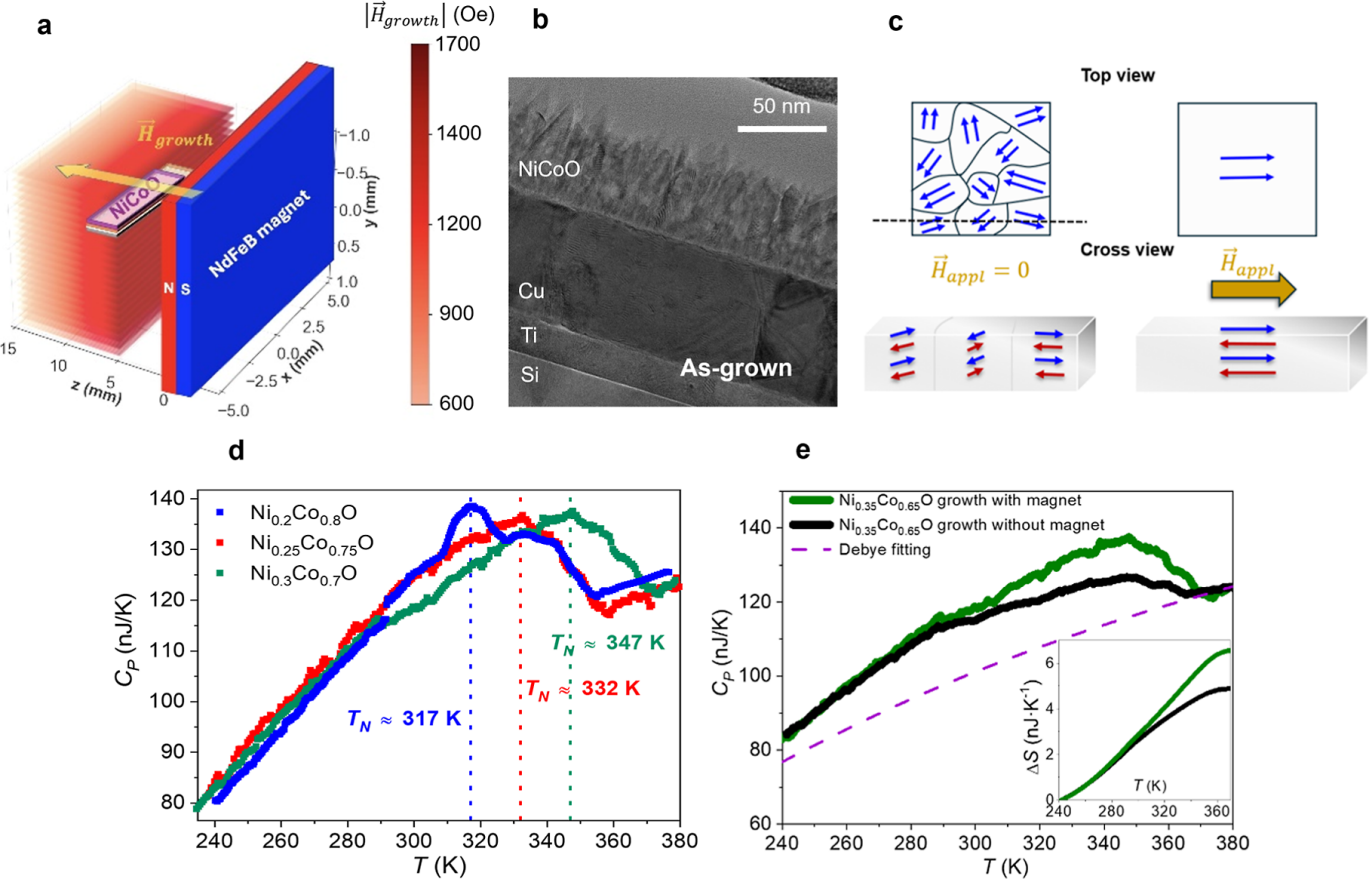
Growth of domain-oriented AFM Ni_*x*_Co_1‑*x*_O (where *x* = 0.2, 0.25, 0.35 and 0.4) films. (a) Distribution of the magnetic field generated by the NdFeB magnet, *H*_growth_. See Methods section for details of the calculations. (b) Transmission electron microscopy (TEM) image of the cross section of an as-grown Ni_0.25_Co_0.75_O film showing its columnar-like structure. (c) Schematics evidencing the role of *H*_growth_ while deposition in the AFM domain configuration achieved in the NiCoO films. (d) Heat capacity as a function of temperature measured for Ni_*x*_Co_1‑*x*_O films with different Ni/Co ratios. (e) Heat capacity as a function of temperature for a Ni_0.35_Co_0.65_O film grown with and without applied magnetic field during sputtering. The blue dashed line represents the Debye fitting used to subtract the phononic contribution (see Methods for further details). The inset displays the accumulated entropy variation associated with the order–disorder transition between 240 and 370 K.

Figure 2a schematically illustrates the electrolyte-cell operating in capacitor-like configuration used to gate the films (see Methods for further details). In our device configuration, the gating voltage is defined with respect to the top Pt foil, while the Cu layer serves as the working electrode. Within this convention, applying a negative voltage drives O^2–^ ions out of the NiCoO layer toward the electrolyte, effectively reducing the oxide. Conversely, a positive gating voltage promotes oxygen incorporation into the NiCoO layer, corresponding to oxidation. All as-grown films are virtually nonferromagnetic, consistent with the formation of AFM phases, as evidenced by the magnetization (*M*) vs. applied magnetic field (*H*) measurement recorded by vibrating sample magnetometry (VSM) for an as-grown Ni_0.35_Co_0.65_O film (Figure S2a). No measurable shift along the easy axis was observed, confirming the absence of EB prior to voltage actuation. The small residual magnetic signal detected in the as-grown state is therefore attributed to trace Fe impurities in the substrate and/or tiny off-stoichiometric regions within the film. *M*-*H* measurements by VSM were acquired at RT and with the applied magnetic field *H* parallel to *H*_growth_ (i.e., parallel to the spin arrangement of the AFM films, denoted as 0° in Figure 2). Ni_*x*_Co_1‑*x*_O films (with *x* = 0, 0.2, 0.25, 0.35, and 0.4) were investigated upon negative biasing at −80 V for 10 min (Figure 2c). These results evidence that the voltage actuation leads to the nonvolatile generation of magnetization ascribed to the partial and permanent reduction of AFM NiCoO to FM NiCo. With increasing Ni content in Ni_*x*_Co_1‑*x*_O, the magneto-ionically induced saturation magnetization (*M*_S_) decreases, consistent with weakened magneto-ionic effects, since Ni hinders voltage-driven oxygen migration.^32^ Except for CoO and the ternary film with the highest Ni content, the remaining ternary films display a shift along the applied magnetic field (i.e., an exchange bias shift, *H*_EB_), demonstrating the effective coupling between the voltage-induced FM phase and the nontransformed (i.e., remaining) AFM matrix. The absence of a shift in CoO arises from its *T*_N_ being 290 K, which suppress AFM order at RT. To exclude possible measurement artifacts, the samples were rotated by 180° and remeasured (blue font data in Figure 2d–h). The observed loop shifts reverse direction, confirming the unidirectional anisotropy induced through FM-AFM coupling. The exchange bias shifts achieved here are comparable to those reported for similar NiCoO-based systems, which are typically realized in Co/NiCoO bilayer structures and require field cooling.^38^ The magnitude of the observed exchange bias is consistent with the intrinsically low magnetocrystalline anisotropy of NiCoO and demonstrates that our approach achieves comparable exchange bias using a single NiCoO antiferromagnetic layer, with the ferromagnetic phase generated internally via magneto-ionic actuation, eliminating the need for an additional ferromagnetic layer or thermal treatment. At higher Ni concentrations (i.e., *x* = 0.4), the magneto-ionic response is almost completely suppressed, resulting in negligible loop shifts. This suppression is attributed to reduced oxygen mobility in Ni-rich environments, which hinders ion migration and prevents the voltage-driven reduction of NiCoO to NiCo. In Figure 2i, the coercivity (*H*_C_) of the films under gating is plotted as a function of Ni content. Unlike CoNi alloys, where increasing Ni content generally leads to a reduction in *H*_C_,^39^ here coercivity increases with Ni concentration. This further confirms the occurrence of FM-AFM coupling which besides inducing a loop shift, it typically leads to a coercivity enhancement.^20,22^ Overall, these results demonstrate that exchange bias can be realized without field cooling, by applying a magnetic field during growth and subsequent voltage gating. As shown in Figure S2a,b, a ternary Ni_0.35_Co_0.65_O film grown without a magnetic field and subsequently actuated by voltage develops a ferromagnetic phase that is not coupled to the remaining AFM, as evidenced by the absence of a shift in the hysteresis loop, confirming the essential role of *H*_growth_ in achieving EB by magneto-ionic means.

**Figure 2:**
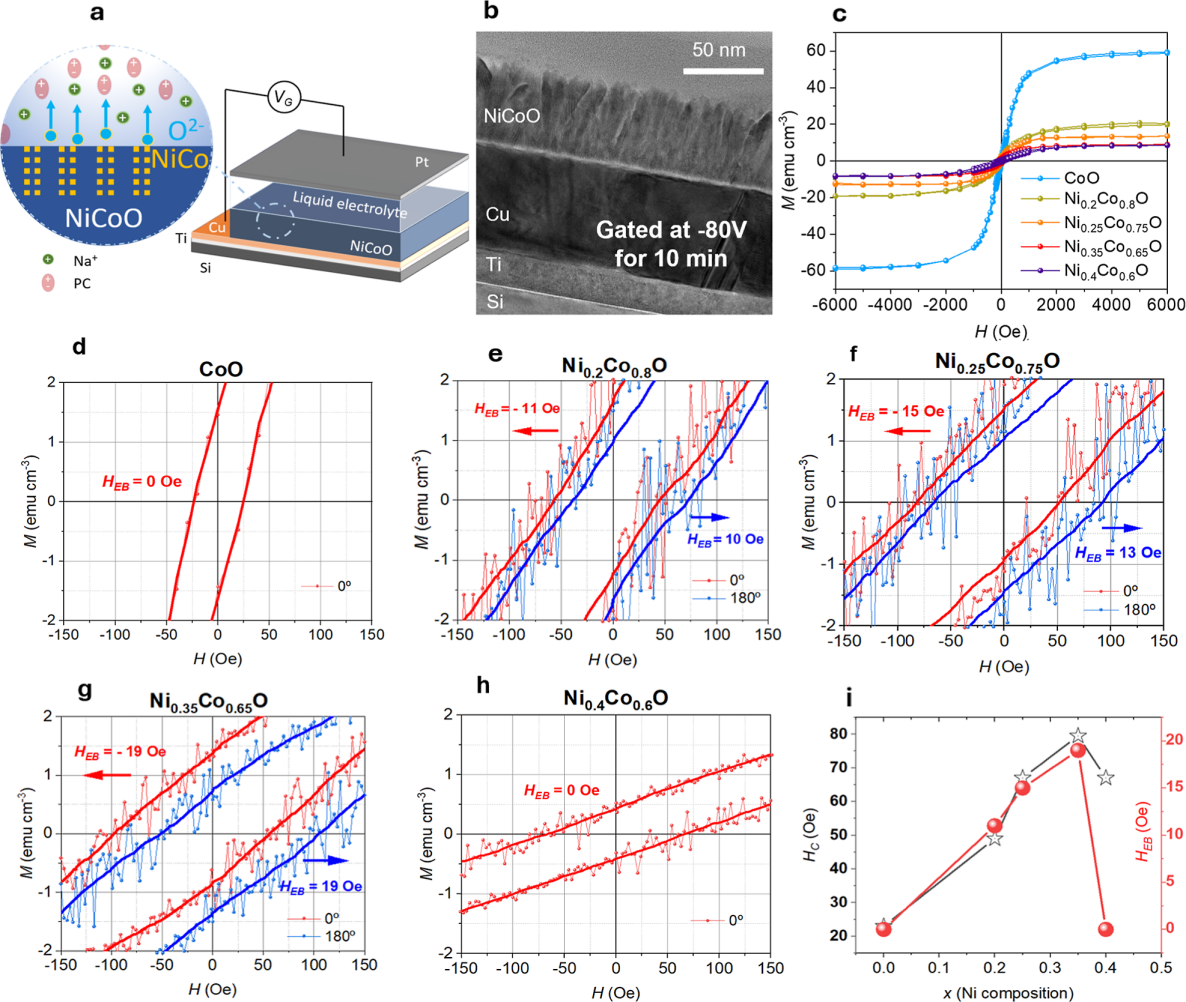
Voltage-driven generation and control of EB in Ni_*x*_Co_1‑*x*_O (where *x* = 0.2, 0.25, 0.35 and 0.4) after −80 V for 10 min. (a) Schematic of the liquid-electrolyte-gating process. The zoom highlights the voltage-induced oxygen ion migration and the subsequent formation of NiCo regions within the NiCoO. (b) Cross-sectional TEM image of a Ni_0.25_Co_0.75_O film after gating at −80 V for 10 min. (c) Room-temperature *M*-*H* loops of the series of Ni_*x*_Co_1‑*x*_O films after being gated at −80 V for 10 min. Panels (d–h) show zoomed loops measured parallel (0°) and antiparallel (180°) to the applied magnetic field used during growth. Solid lines are smoothed curves superimposed on the raw data for clarity. (i) Evolution of EB shift, *H*_EB_, and coercivity, *H*_C_, as a function of the amount of Ni, *x*. The lines are guides to the eye.

Additionally, VSM measurements were performed at low temperature along the field-cooling direction on a 50 nm-thick CoO film gated at −80 V for 10 min. The sample was field-cooled from room temperature to 130 K under 10 kOe, and *M*-*H* loops were recorded upon heating in 20 K steps up to 290 K. Pronounced exchange-bias shifts (*H*_EB_) are observed at low temperature, reaching ≈ −300 Oe at 130 K (Figure S3), together with an increased coercivity reflecting stronger pinning of magneto-ionically generated ferromagnetic Co clusters by the antiferromagnetic CoO matrix. The magnitude of *H*_EB_ decreases with increasing temperature and vanishes at 290 K, corresponding to the Néel temperature of CoO. Below this temperature, CoO exhibits a significantly larger exchange bias than NiCoO, consistent with the higher antiferromagnetic anisotropy of CoO and the resulting stronger interfacial spin pinning.^20^

For all magneto-ionic films (i.e., Ni_*x*_Co_1‑*x*_O films with *x* = 0.2, 0.25, and 0.35), the voltage-induced magnetization remains relatively small, indicating that only a limited amount of NiCo is formed, part of which likely exists in a superparamagnetic state, as suggested by the S-shaped hysteresis loops of the ternary films (Figure 2).

**Figure 3:**
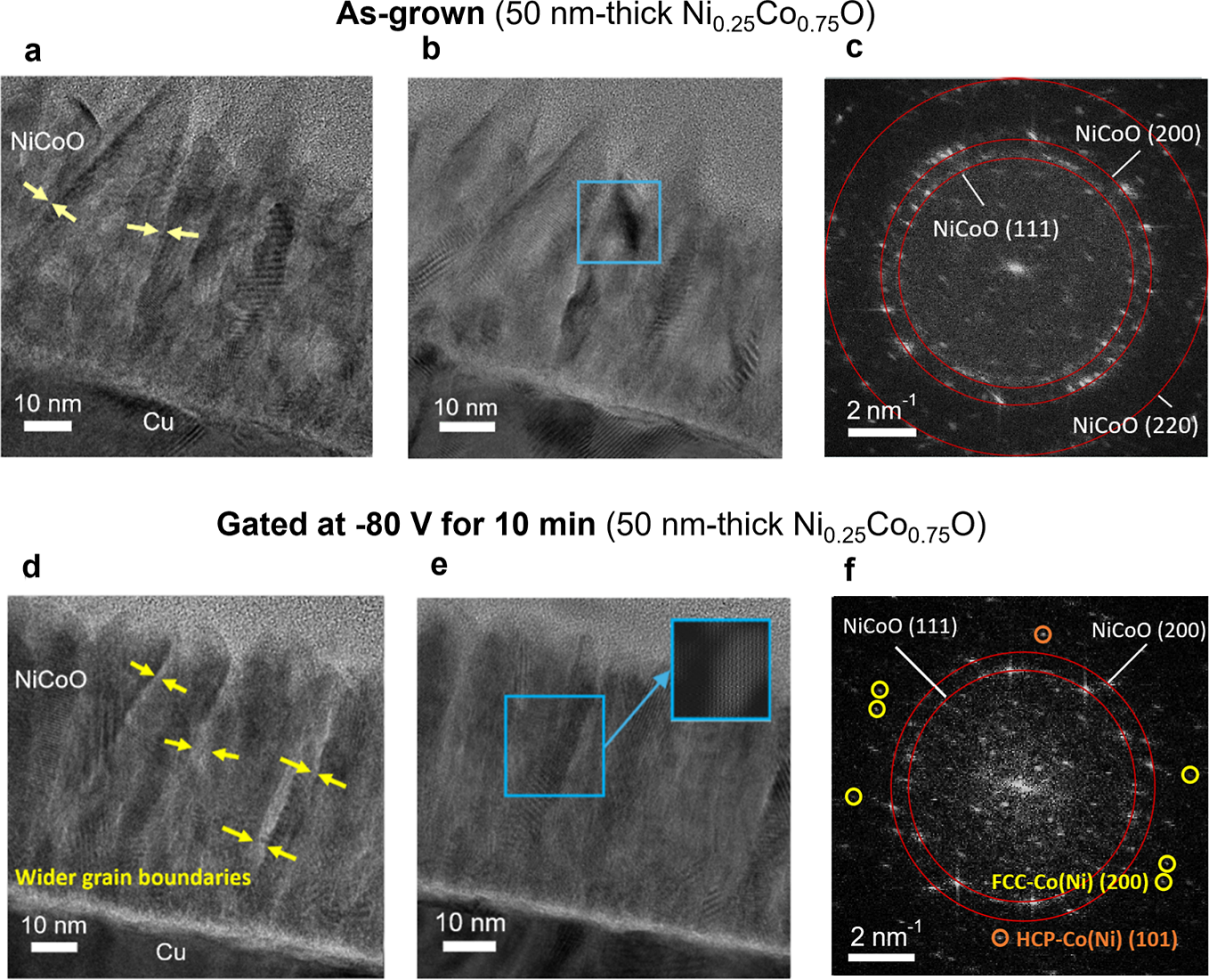
TEM characterization of the cross-section of an as-grown Ni_0.25_Co_0.75_O film and a Ni_0.25_Co_0.75_O film actuated with −80 V for 10 min (a,b) Cross-sectional TEM images of an as-grown Ni_0.25_Co_0.75_O film acquired in two different regions. (c) FFT of the region marked with a blue square in panel (b). (d,e) Cross-sectional TEM images of a Ni_0.25_Co_0.75_O film acquired in two similar regions after applying −80 V for 10 min (f) FFT of the region indicated by a blue square in panel (e). Spots from metallic clusters are highlighted. Tables S1 and S2 show the interplanar distances from the spots of panels (c) and (f), respectively.

Transmission electron microscopy (TEM) characterization of the cross sections of an as-grown and a voltage-gated Ni_0.25_Co_0.75_O film was carried out to investigate the impact of gating on the microstructure. A comparative analysis of Figure 3a,b (as-grown) vs. Figure 3d,e (gated at −80 V for 10 min) reveals that voltage actuation results in broadened grain boundaries, particularly in the upper part of the film, where the columnar grains are more well-defined. As shown by the fast Fourier transform (FFT) analyses (Figure 3c,f) of the blue-marked regions in the high-resolution TEM images of Figure 3b,e, all diffraction spots in the as-grown film are consistent with NiCoO (Table S1). However, after gating, new diffraction spots emerge, assigned to HCP-Co­(Ni) with an interplanar spacing of 1.88 Å (corresponding to the (101) planes, PDF 00-005-0727) and FCC-Co­(Ni) with *a* spacing of 1.73 Å (corresponding to the (200) planes, PDF 00-015-0806). This confirms that NiCoO undergoes voltage-driven reduction to metallic NiCo phases (Table S2). Additionally, with the goal of locating the voltage-induced FM regions, a detailed high-resolution TEM characterization was carried out next to the grain boundaries (Figure S4), which are known to act as diffusion channels for ion migration.^3^ As seen in Figure S4, metallic regions tend to form discontinuously along the grain boundaries.

Figure 4 shows the results of variable energy positron annihilation lifetime spectroscopy (VEPALS), used to probe the evolution of structural defects in terms of size and density upon gating −80 V for 2 h. The defect size and density are characterized by the positron lifetime (τ) and relative intensity (*I*), respectively (see Methods). τ_1_ and τ_2_ correspond to vacancy complexes located within the grains and grain boundaries, respectively.^40^ For this analysis, a thicker Ni_0.2_Co_0.8_O film (200 nm) was grown to achieve suitable depth resolution. As shown in Figure 4a, variations in defect size and density primarily occur in the upper region of the film, as magneto-ionic activity initiates at the surface and progresses downward. In both as-grown and voltage-gated films, grain boundaries dominate over vacancy clusters, as indicated by the *I*_2_ values exceeding 50%. Remarkably, upon gating, grain boundaries broaden (larger τ_2_) but decrease in fraction (lower *I*_2_), in agreement with the TEM observations. The average defect size (τ_av_ in Figure S5) remains nearly unchanged, as the size of small vacancy clusters (τ_1_) decreases and their concentration (*I*_1_) rises. However, based on Doppler broadening variable energy positron annihilation spectroscopy (DB-VEPAS) measurements (see Methods), a large increase of the *S*-parameter, typically scaling with defect concentration, is found for the gated film (see Figure S6a). Such behavior can be only explained by the concomitant change in the local chemistry at the annihilation sites, here to a larger extent at grain boundaries (as seen from τ_2_ and *I*_2_). Every material has its characteristic *S* and *W* fractions, whereas *S* carries information about defect density and size.^41^ Gating induces a shift to lower *W*-fraction values, consistent with the formation of NiCo-rich configurations at the grain boundaries (Figure S6b). That tendency is significant in the top part of the film, whereas the interface region remains more stoichiometric. This agrees with the broadening of grain boundaries due to voltage-driven oxygen migration, which induces partial reduction of adjacent NiCoO to NiCo, as observed in 50 nm-thick Ni_0.2_Co_0.8_O films.

**Figure 4:**
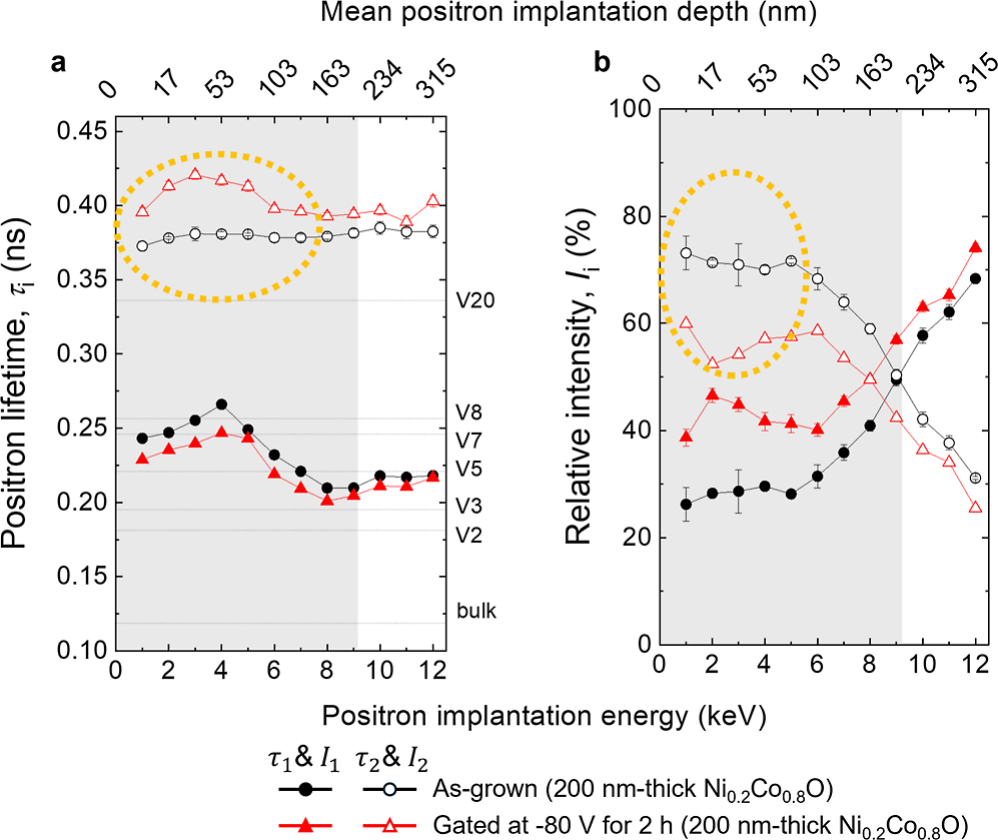
Structural defect characterization by VEPALS (a) Positron lifetime components (i.e., τ_1_ and τ_2_): and (b) the corresponding relative intensities (i.e., *I*_1_ and *I*_2_) as a function of positron implantation energy (or depth) of as-grown and gated (−80 V for 2 h) 200 nm-thick Ni_0.2_Co_0.8_O films. τ_1_ and τ_2_ components represent respectively small and large voids. τ_2_ is the majority defect in the top part of the film. After gating, τ_2_ increases, consistent with the enlargement of grain boundaries (i.e., ionic channels) in the upper region of the material. Conversely *I*_2_ decreases indicating the number of large voids drops. “V#” stands for the number of vacancies based on DFT calculations for Co_3_O_4_.^40^

TEM, VEPALS, and magnetometry (through the characteristic S-shaped hysteresis loops) reveal that voltage induces the formation of small FM regions, preferentially located alongside the grain boundaries in a discontinuous manner. To better understand the microscopic origin of the induced EB, we carried out micromagnetic simulations designed to probe the role of AFM interfacial uncompensated spins (UCSs), known to be crucial for EB, in NiCoO.^20^ The model^42^ enables EB simulations in polycrystalline systems with vertical exchange interactions and multiple NiCo/NiCoO interfaces (see Methods). In this framework, NiCo (FM) grains are embedded within a continuous NiCoO (AFM) matrix, reproducing the experimental microstructure (3 FM grains out of 255 ≈ 1%, see Methods for the justification). The simulated hysteresis loops were compared with experimental data for Ni_0.35_Co_0.65_O, chosen as a representative composition due to its largest EB shift (Figure S7). The modeling was progressively refined by introducing additional degrees of freedom. In the baseline case (Figure S7a), only the FM grains are considered, without AFM UCSs. Because of the small FM fraction, a stochastic thermal field was added to reproduce thermal fluctuations at 300 K, accounting for the superparamagnetic-like response of the FM phase. As expected, without exchange coupling, coercivity and remanence are negligible. When set-type AFM UCSs (pinned spins, see Methods) are included (Figure S7b), EB appears due to interfacial exchange interactions, but coercivity remains essentially unchanged. This shows that set-type UCSs alone cannot explain the experimentally observed coercivity enhancement. In contrast, when both set- and rot-type UCSs (rotatable spins, see Methods) are incorporated (Figure S7c), coercivity increases significantly, and the simulated loop closely resembles experimental results. At this stage, UCSs are assumed to be unaffected by Zeeman and demagnetizing fields, consistent with conventional EB models in layered systems. While EB is reproduced under this assumption, the loop tilt and field-dependent features deviate from experiment. Further refinement (Figure S7d) incorporates the effect of Zeeman and demagnetizing fields into the effective field (***H***_**eff**, ***i***_) acting on each UCS. This is motivated by both the large amount of UCSs (3 times the number of FM grains) and their ferromagnetically like behavior (see Methods). This approach yields the best agreement with experimental hysteresis loops, accurately reproducing the EB, coercivity, and loop tilt. The improvement is consistent with the presence of discontinuous FM regions and suggests that UCSs in this system behave in a more active, FM-like manner than typically assumed in EB bilayers, undergoing demagnetizing-like effects, in concordance with the existence of isolated FM counterparts, evidenced by TEM (Figure S4). Overall, these results demonstrate that, in our magneto-ionic films, EB does not primarily stem from the small voltage-induced NiCo fraction. Instead, it is governed by interfacial UCSs, which strongly couple to the FM grains and dictate the macroscopic hysteresis behavior.

To further confirm the coupling between the magneto-ionically generated FM clusters and the remaining AFM matrix, we investigated the magnetic anisotropy of the ternary film exhibiting the largest exchange bias (i.e., Ni_0.35_Co_0.65_O) using in plane configuration ferromagnetic resonance (FMR). In these measurements, the applied magnetic field *H*_FMR_ was oriented either parallel or perpendicular to *H*_growth_ depending on the sample orientation: 0°, where the resonance field (*H*_FMR_) is parallel to the deposition field (*H*_growth_ in Figure 1c), and 90°, where it is perpendicular (see Figure S8). A clear anisotropy is observed in our system, as the resonance FMR peak intensity along the easy axis (0°) is stronger and better defined than along the hard axis (90°). The FMR peak allows us to obtain the resonance field for the multiple frequencies. This confirms the presence of unidirectional anisotropy, induced by exchange coupling between the magneto-ionically generated NiCo clusters and the preoriented NiCoO matrix deposited under an applied magnetic field (Figure S8). This is in full agreement with the micromagnetic simulations, which demonstrate that exchange bias and coercivity enhancement originate from the interaction of FM regions with interfacial UCSs. The FMR anisotropy reveals that such coupling is directionally locked to the growth field, supporting the interpretation that UCSs are oriented during deposition and subsequently couple to the voltage-induced FM fraction. Together, the micromagnetic simulations and FMR measurements provide a coherent picture: EB in magneto-ionic films does not primarily arise from the small fraction of NiCo formed, but instead from the dominant role of interfacial UCSs, whose orientation and coupling are governed by the field-assisted sputtering deposition. Additional measurements consistent with the FMR characterization are provided by hysteresis loops recorded with the magnetic field applied parallel (0°) and perpendicular (90°) to the growth-field direction for the Ni_0.35_Co_0.65_O film gated at −80 V for 10 min (Figure S8c). When measured along 0°, the loop is shifted and becomes more square-shaped, with larger remanent magnetization and coercivity, indicating that this direction corresponds to the magnetic easy axis established by exchange-bias coupling. In contrast, the loop measured at 90° shows no bias shift and exhibits reduced remanence and coercivity, characteristic of a hard-axis magnetic response.

To explore not only the voltage-controlled generation of exchange bias but also its modulation, a series of Ni_0.35_Co_0.65_O films was subjected to varying gating time of 5, 10, and 30 min while applying −80 V (Figure 5). The results reveal a clear dependence of EB on the duration of the applied bias. The highest EB, of around 22 Oe, is observed after 5 min of gating. This behavior is attributed to the formation of smaller CoNi clusters, analogous to thinner FM layer in a FM/AFM bilayer system and hence resulting in enlarged exchange bias shift since *H*_EB_ is inversely proportional to the thickness of the FM counterpart.^20^ As the ferromagnetic part becomes thicker, the same interfacial exchange energy is distributed over a larger magnetic moment, reducing the effective bias field. At longer gating times (e.g., 30 min), NiCo clusters grow in size, consequently reducing the exchange bias shift. This leads to a diminished EB effect (Figure 5e). Furthermore, larger ferromagnetic regions favor domain formation and nonuniform spin rotation, which further weakens the loop shift.^20^ Importantly, the EB field is largely reversible in our system, as demonstrated in Figure S2c. Applying −80 V for 10 min induces a clear EB, while reversing the voltage polarity for an additional 5 min nearly suppresses the magnetization, highlighting the voltage-programmable nature of EB in these films.

**Figure 5:**
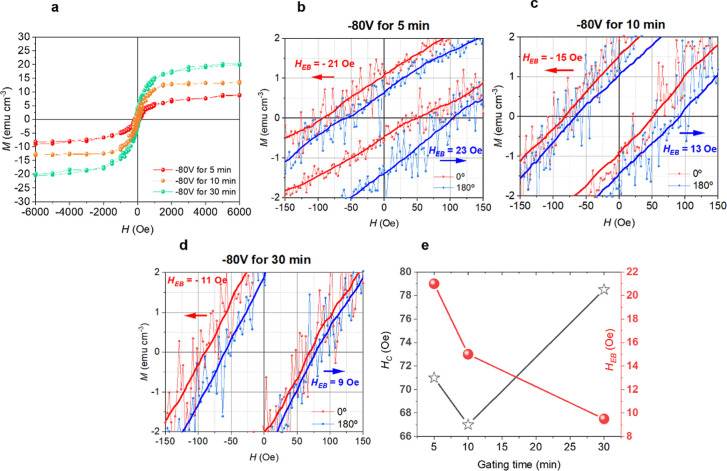
Voltage-driven modulation of the EB in a Ni_0.25_Co_0.75_film gated for different times. (a) *M*-*H* measurements of Ni_0.25_Co_0.75_ films upon applying −80 V for 5, 10, and 30 min (b–d) Zoomed hysteresis loops corresponding to Ni_0.25_Co_0.75_ films upon applying −80 V for 5, 10, and 30 min, respectively, measured parallel (0°) and antiparallel (180°) to the applied magnetic field used during growth, *H*_growth_. Solid lines are smoothed curves superimposed on the raw data for clarity. (e) Exchange bias shift (*H*_EB_) and coercivity (*H*_C_) as a function of gating time.

Remarkably, the induced magnetization can be mainly reversed by applying a voltage protocol of opposite sign (Figure S2c), which drives the oxidation of the FM regions.

The onset voltage required to induce magneto-ionic effects in films with lower Ni content (i.e., Ni_0.2_Co_0.8_O and Ni_0.25_Co_0.75_O) lies between −40 and −60 V for gating times on the order of 10 min. Because the effects observed at −60 V were relatively weak, a voltage-gating amplitude of −80 V was selected to ensure pronounced magneto-ionic effects across all Ni compositions. This choice also enables measurable magneto-ionic response within gating times below 5 min for all films, noting that the magneto-ionic response weakens with increasing Ni content.^32^ The dependence of the exchange bias shift on the voltage-gating amplitude is presented in Figure 6. While the saturation magnetization increases in magnitude with increasing voltage-gating amplitude due to the formation of ferromagnetic NiCo, the exchange bias shift decreases. This behavior is consistent with the growth of the ferromagnetic phase, which leads to a reduction in the exchange bias field.^20^

**Figure 6:**
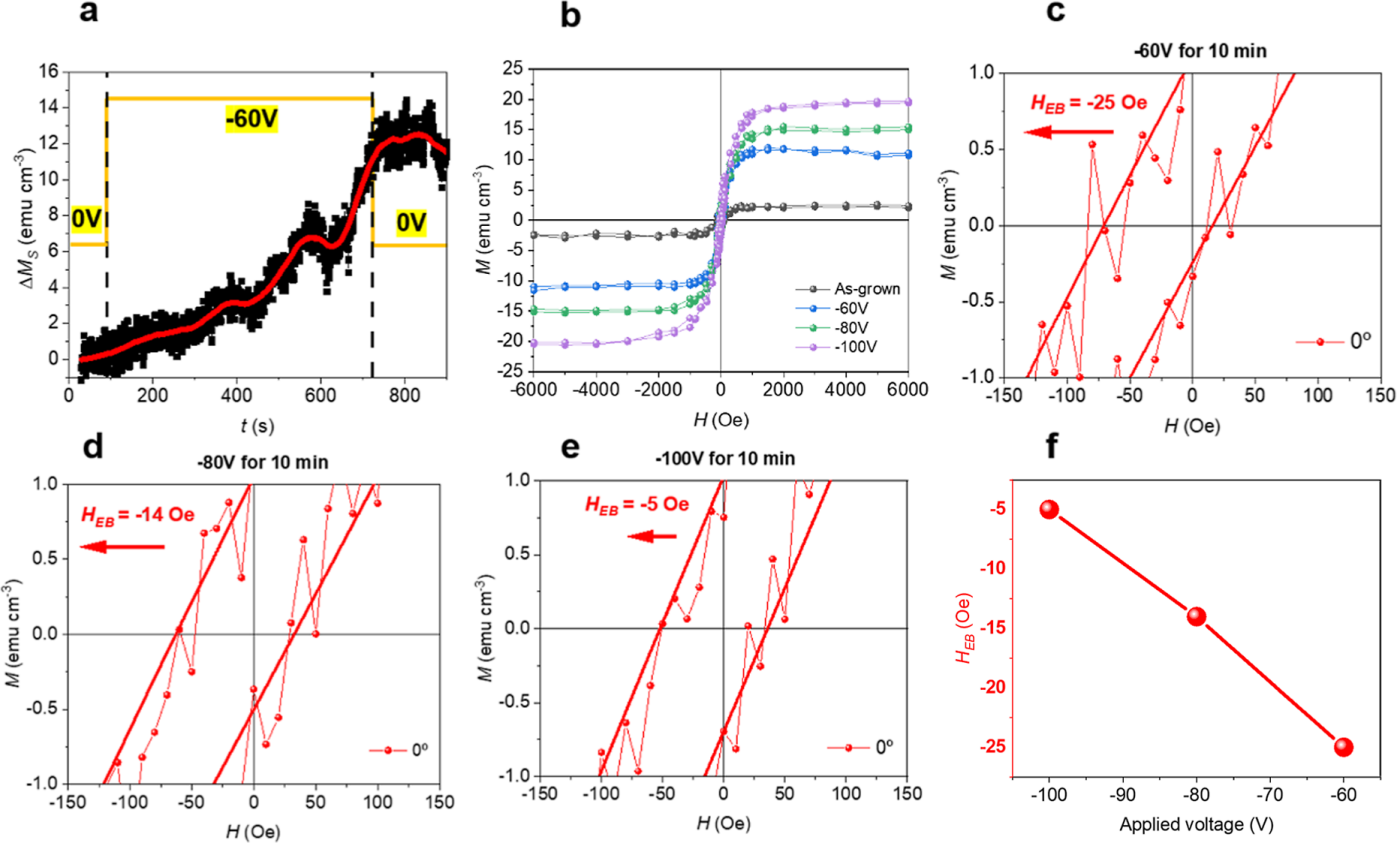
EB programmability through voltage-gating amplitude in Ni_0.25_Co_0.75_O films. (a) Time-dependent magnetization measurement under an applied magnetic field of 10 kOe while applying −60 V. (b) Hysteresis loops of Ni_0.25_Co_0.75_O films upon being gated at −60, −80, and −100 V for 10 min (c-e) Zoom-in of panel (b) highlighting the coercive-field region for the films gated at −60, −80, and −100 V for 10 min, respectively. Solid lines are smoothed curves superimposed on the raw data for clarity. (f) Exchange bias shift (*H*_EB_) as a function of gating voltage.

To validate programmability through cyclability, we performed endurance tests on a Ni_0.25_Co_0.75_O film (Figure 7) using a sequence of five voltage pulses (−80 V/+80 V, 10 and 15 min, respectively). After the fifth cycle, a final −80 V pulse was applied for 10 min and the voltage was then switched off. Hysteresis loops were subsequently recorded with the applied magnetic field parallel (0°) and antiparallel (180°) to the growth-field direction. As shown in Figure 7b, the hysteresis loops exhibit opposite shifts, with exchange-bias shifts slightly larger than those obtained after a single −80 V pulse of 10 min (Figure 2f). The increased shift results from the system not fully recovering its initial state upon cycling, as indicated by the reduced saturation magnetization. This decrease suggests smaller FM clusters, which in turn slightly enhance the exchange bias magnitude. However, since the increase is relatively small, it indicates that the uncompensated AFM spin configuration remains rather unchanged upon cycling.

**Figure 7:**
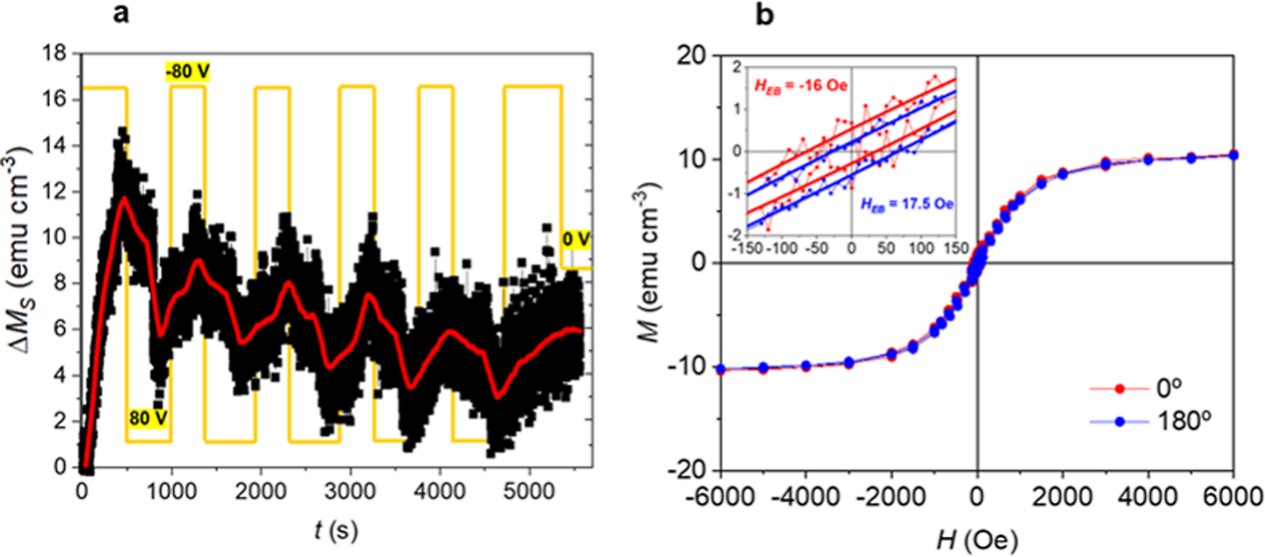
Endurance. (a) Generated saturation magnetization (Δ*M*_S_) as a function of time in a 50 nm-thick Ni_0.25_Co_0.75_O film during the application of a sequence of five voltage pulses (−80 V/+80 V, with durations of 10 and 15 min, respectively), followed by a final −80 V pulse of 10 min before switching off the voltage. Magnetometry was performed under an applied magnetic field of 10 kOe to ensure full saturation of the magneto-ionically generated ferromagnetic component. (b) Room-temperature magnetization (*M*) vs. applied magnetic field (*H*) measurements after the five voltage-cycling steps and following a sixth −80 V pulse, measured parallel (0°) and antiparallel (180°) to the applied magnetic field used during growth, *H*_growth_. The inset shows a zoom-in of the coercive-field region.

As mentioned before, voltage-induced EB appears only in films grown under an applied magnetic field. In contrast, samples deposited without a field show no EB, even though they form similar voltage-induced FM NiCo clusters (Figure S2a,b). This highlights the essential role of AFM spin alignment during growth under an applied magnetic field, which effectively replaces the conventional field-cooling process by imprinting a unidirectional anisotropy in the AFM layer at RT. When the AFM film is grown without a magnetic field, multiple AFM domains with no-preferential orientation form, preventing a net coupling with the subsequent voltage-induced FM regions and thus hindering the exchange bias.

It is worth noting that the data shown in the manuscript correspond to one of several independent measurements, all of which exhibit the same qualitative and quantitative trends in coercivity and exchange bias shift as functions of the Ni/Co ratio, gating time, and voltage-gating amplitude. Consistent exchange bias behavior is also observed in the films grown for VEPALS characterization. Since VEPALS requires both sufficiently thick films to ensure representative depth resolution and large sample areas to achieve an adequate signal-to-noise ratio, thicker (200 nm) Ni_0.2_Co_0.8_O films were grown and gated at −80 V for longer durations (2 h) to obtain saturation magnetization values comparable to those of thinner films, as increased thickness weakens magneto-ionic effects. These samples exhibit an improved signal-to-noise ratio due to the larger magnetic volume arising from both the increased sample area and film thickness (Figure S9). The increased coercivity relative to the 50 nm films arises from thickness-induced changes in crystallinity, grain size, texture, and surface morphology. In addition, measurements from an independent replica of the 50 nm-thick Ni_0.25_Co_0.75_O film show an exchange bias shift of ≈15 Oe (Figure S10), in excellent agreement with the values reported in Figure 2f.

While the present implementation can be still improved in terms of energy-efficiency, this work should be regarded as a proof-of-principle demonstrating the potential of magneto-ionic control without thermal treatment or Joule heating. The relatively high gate voltages (±80 V) and long gating durations (5–30 min) were intentionally employed to produce clearly detectable magnetic changes. Note that a significant fraction of the applied voltage drops across the dielectric rather than the active magneto-ionic film. Hence, energy efficiency could be substantially improved through optimized electrolyte conductivity; for example, the addition of 10^–4^ M KI in propylene carbonate has been shown to reduce the required actuation voltage by approximately 1 order of magnitude.^43^

## Conclusions

We demonstrate the magneto-ionic generation of EB at RT in 50 nm-thick AFM NiCoO single films, achieved without the conventional requirement of field cooling. By having a magnetic field during film growth, the antiferromagnetic uncompensated spins become preferentially aligned, producing a nearly oriented AFM matrix. This controlled alignment provides the foundation for robust EB effects once the NiCo FM counterpart is formed by magneto-ionic means. Application of a gating voltage partially reduces NiCoO, driving the formation of ferromagnetic NiCo clusters discontinuously along the vertical grain boundaries of the columnar AFM NiCoO matrix. TEM and VEPALS analyses confirm directly and indirectly, respectively, the emergence of these nanoscale FM regions. This electrochemical process is largely reversible, enabling dynamic control over EB within the same AFM layer. Importantly, the EB magnitude can be modulated through three independent parameters. First, increasing the Ni content in Ni_*x*_Co_1‑*x*_O raises the Néel temperature, thereby strengthening the unidirectional anisotropy and resulting in exchange bias shifts of up to ≈25 Oe. Second, varying the actuation time modulates the size of the voltage-induced ferromagnetic NiCo clusters: longer actuation times promote cluster growth and consequently weaken the exchange bias, reflecting the time-dependent nature of the magneto-ionic process. Third, adjusting the voltage amplitude similarly controls the size of the voltage-induced ferromagnetic NiCo clusters, with larger absolute gating voltages favoring cluster growth and leading to a corresponding reduction in exchange bias. Micromagnetic simulations reveal that EB in this system is governed by the interfacial uncompensated spins of the AFM matrix, which couple strongly to the embedded FM clusters and dominate the macroscopic hysteresis response. This unconventional mechanism highlights that EB arises not merely from FM inclusions but from the active role of interfacial spins in a polycrystalline AFM network. These results constitute a proof-of-principle demonstration of voltage-controlled exchange bias in a magneto-ionically active, single antiferromagnetic layer, representing a realization of exchange bias achieved solely through electrical control and without thermal assistance. Although the present NiCoO system is not optimized for endurance, it establishes a conceptual framework for future material platforms. In this regard, antiferromagnetic oxides and nitrides with higher ion mobility and faster electrochemical kinetics, such as LaFeO_3_ ^44^ and CoMnN,^45^ are promising candidates. This work thus establishes a paradigm for fully electrically programmable EB, enabling nonvolatile and secure information encoding by leveraging both the intrinsic magnetic anisotropy of the AFM and the reconfigurable nature of the magneto-ionically generated FM regions within the AFM single layer. This approach offers a simple, energy-efficient route toward next-generation AFM spintronic devices.

## Methods

### Sample Preparation

50 nm nominal thickness of Ni_*x*_Co_1‑*x*_O films were grown at room-temperature by DC reactive magnetron cosputtering with Ni and Co metallic targets on top of Si (100) substrates, previously coated with Ti (20 nm) and Cu (60 nm) layers that serve as working electrode. An AJA International ATC 2000 V ultrahigh vacuum sputtering system with a base pressure of around 3 × 10^–8^ Torr and a working pressure of 3 mTorr was used in an Ar and O_2_ gas mixture atmosphere. The Ar/O_2_ flow ratio was set at 9% O_2_ partial pressure to obtain the rock salt CoO rather than the spinel phase Co_3_O_4_.^46^ The distance between the substrate and targets was 10 cm. A series of samples were prepared by varying the deposition power for Ni to 0, 15, 20, 25, and 30 W, resulting respectively in CoO, Ni_0.2_Co_0.8_O, Ni_0.25_Co_0.75_O, Ni_0.35_Co_0.65_O and Ni_0.4_Co_0.6_O. The deposition power of Co was fixed to 50 W. During the deposition of Ni_*x*_Co_1‑*x*_O films, an external magnetic field (*H*_growth_) was applied by placing a NdFeB permanent magnet next to the samples. *H*_growth_ was estimated by analytically computing the magnetic field H⃗(r⃗) generated by a uniformly magnetized permanent magnet with rectangular-prism geometry in vacuum, within the magnetostatic approximation. The magnetic field outside the magnet was obtained using the standard magnetostatic formulation based on equivalent magnetic surface charge densities located on the faces perpendicular to the magnetization direction.^47^ The magnet dimensions were 40 mm × 20 mm × 5 mm along the *x*, *y*, and *z* axes, respectively. A uniform magnetization $\mathrm{M⃗}=M{\mathrm{e⃗}}_{z}$ was assumed, with M = 1145.2 emu·cm^–3^, corresponding to the saturation magnetization of the NdFeB permanent magnet used. The origin of the Cartesian coordinate system was placed at the center of the upper surface of the magnet, along the *z* axis. The magnetic field was evaluated within the region of interest defined by −5 mm ≤ *x* ≤ 5 mm, −1 mm ≤ *y* ≤ 1 mm, and 5 mm ≤ *z* ≤ 15 mm.

### Structural Characterization by X-ray Diffraction (XRD) and Transmission Electron Microscopy (TEM)

θ/2θ XRD patterns were acquired using a Malvern-Panalytical X’Pert Pro MRD system with Cu Kα radiation.

TEM was carried out on a Spectra 300 (S)­TEM microscope (Thermo Fisher Scientific), operated at 200 kV. Prior to TEM observations, cross-sectional lamellae were prepared by focused ion beam, placed onto a copper transmission electron microscopy grid, and sputter-coated with a protective platinum layer.

### Compositional Characterization by Energy Dispersive X-ray (EDX) Analysis

The composition of the Ni_*x*_Co_1‑*x*_O films was determined by EDX analysis.

### Magneto-Electric Characterization

To explore the magnetic response of Ni_*x*_Co_1‑*x*_O films under voltage actuation, we used a custom-designed electrolytic cell in capacitor-like configuration, compatible with the vibrating sample magnetometer (VSM) (Figure 2a). The measurements were performed along the plane of the films and gate voltages were applied while carrying the magnetic measurements. Room-temperature magnetization loops were recorded under in-plane magnetic fields up to 1 T using a VSM from MicroSense (LOT, Quantum Design). Voltage actuation was achieved by applying a potential difference between the counter electrode (a Pt sheet) and the Cu working electrode using an external power supply (Agilent B2902A). The liquid electrolyte consisted of anhydrous propylene carbonate, a polar aprotic solvent, containing solvated Na^+^ and trace OH^–^ species (≈30 ppm) to enhance ionic conductivity.

### Microsecond-Pulsed Heating Nanocalorimetry

Heat-capacity of the Ni_*x*_Co_1‑*x*_O films was characterized by microsecond-pulsed heating nanocalorimetry over the temperature range of 230–380 K. A differential setup was employed using a custom-fabricated twin nanocalorimeter in series configuration: one calorimeter served as the reference to subtract background contributions and the other with the film directly deposited on the sensing membrane, without the use of a mask thereby simplifying the fabrication process. This is one of the main advantages of using this system instead of quasi-adiabatic nanocalorimetry.^37,48^ Measurements were conducted under high vacuum inside a cryogenic environment to minimize heat losses. The heat capacity Δ*C*_P_(*T*_S_(*t*)) of the film was extracted from the differential response between the sample (*S*) and the reference (*R*) using the following formula

1 $${\Delta C}_{P}\left(T_{S}\left(t\right)\right)=\frac{I\Delta V}{\beta_{S}}-\frac{V_{R}}{\beta_{S}\beta_{R}}\frac{{\left(d\Delta V/dt\right)}_{t}}{{\left(dR_{S}/dT_{S}\right)}_{t}}+\frac{V_{R}I}{\beta_{S}}\left[1-\frac{{\left(dR_{R}/dT_{R}\right)}_{t}}{{\left(dR_{S}/dT_{S}\right)}_{t}}\right]$$

The full derivation of the differential heat capacity can be found in.^48^

To extract the magnetic contribution to the heat capacity, the entropy change (Δ*S*) is determined from the temperature integral of the heat capacity divided by the temperature (*C*_P_/*T*). This procedure requires subtraction of the phononic (lattice) contribution from the experimental *C*_P_(*T*) data. The lattice contribution is modeled according to the Debye expression for the heat capacity, including a proportionality factor *A* to account for deviations arising from the specific characteristics of the measured sample

2 $$C_{P}^{ph}\left(T\right)=A{\left(\frac{T}{T_{D}}\right)}^{3}\int_{0}^{T_{D}/T}\frac{t^{4}e^{t}}{{\left(e^{t}-1\right)}^{2}}dt)$$

*T*_D_ is the Debye temperature, and *A* is a scaling parameter determined from the best fit to the experimental data. Numerical integration of the Debye function enables accurate estimation of the lattice contribution, which is subsequently subtracted from the total *C*_p_(*T*)­to isolate the magnetic entropy associated with the antiferromagnetic transition.

### Micromagnetic Simulations

Micromagnetic simulations^49^ were performed on a mesh of 512 × 512 × 10 magnetic cells, each with a volume of 1 × 1 × 5 nm^3^, to shed light on the origin of exchange bias in Ni_*x*_Co_1‑*x*_O films. Simulations focus on the exchange bias behavior of the Ni_0.35_Co_0.65_O film actuated at −80 V for 10 min since it results in the largest exchange bias shift. Polycrystallinity of the Ni_0.35_Co_0.65_O/CoNi system is introduced by using a 2D Voronoi tessellation, which divides the sample into 255 grains (85 per block) with an average radius of 2 nm. Among these, 3 represent the FM buried grains (approximately 1%), 3 are assigned as set-type UCS, and 9 as rot-type UCS. The remaining grains are modeled as a compensated AFM matrix, which does not contribute to the net magnetization. On average, each FM grain interacts with 1 set-UCS and 3 rot-UCS grains. The set-type UCS grains correspond to pinned spins, whose magnetization remains fixed during FM reversal. In contrast, rot-type UCS grains represent rotatable spins that can be reoriented in response to the local effective field. To avoid finite-size effects, the system was repeated 10 times in both *x* and *y* directions. Others parameters were fixed throughout the simulations: the uniaxial anisotropy constants *K*_FM_ = 4.0 × 10^4^ J/m^3^, *K*_rot_ = 1.75 × 10^5^ J/m^3^, *K*_set_ = 1.4 × 10^6^ J/m^3^ and exchange coupling constants *J*_set_ = 9.3 × 10^–4^ J/m^2^, *J*_rot_ = 4 mJ/m^2^. The superparamagnetic behavior is a result of implementing stochastic thermal field to mimic the thermal fluctuations that occur at a temperature of 300 K. Hysteresis loops are obtained via energy minimization, such that each cell magnetization (***m***_***i***_) is parallel to the effective field (***H***_**eff**, ***i***_). Each magnetic cell behaves as a spin that interacts with its neighbors via an exchange stiffness set uniformly at *A*_ex_ = 3 pJ/m for both FM and AFM grains. ***H***_**eff**, ***i***_ is the sum of different contributions such as anisotropy, demagnetizing and thermal effects. At grain boundaries, no exchange interaction is assumed between FM grains. The only grain interactions considered are the exchange anisotropy between an FM grain and neighboring UCS grains (*set* or *rot*), which adds the following terms to ***H***_**eff**_

3 $$H_{ex,FM}=\frac{2J_{i}}{\mu_{0}\delta M_{FM}}\left(m_{FM}-m_{AFM}\right)$$

4 $$H_{ex,AFM}=\frac{2J_{i}}{\mu_{0}\delta M_{AFM}}\left(m_{AFM}-m_{FM}\right)$$

where μ_0_ is the vacuum permeability and δ is the spatial range where the exchange interaction acts, which is twice the cell size, along the direction of contact. *M*_FM_ and *M*_AFM_ are the FM and AFM saturation magnetizations, respectively which are set as *M*_FM_ = *M*_AFM_ = 1.19 MA/m to ensure consistent energy distribution. This value aligns with the expected contributions of Ni (0.45 MA/m) and Co (1.4 MA/m) at the given concentrations of Ni_0.35_Co_0.65_O sample. *J*_*i*_ represents the exchange coupling constants, *i* denoting *set* or *rot*-type UCS.

### Ferromagnetic Resonance (FMR)

Broadband FMR in the microwave range of 2–20 GHz were measured with a coplanar waveguide ferromagnetic resonance (CPW-FMR) spectrometer setup provided by NanOsc Instruments AB. Measurements are performed at a fixed frequency while sweeping the applied magnetic field *H*_FMR_.

### Variable Energy Positron Annihilation Lifetime Spectroscopy (VEPALS)

Defect characterization was carried out by VEPALS at the Monoenergetic Positron Source (MePS) at Helmholtz-Zentrum Dresden–Rossendorf (Germany).^50^ A CeBr_3_ scintillator detector together with a Hamamatsu R13089-100 photomultiplier tube for the gamma photons detection was employed. A Teledyne SPDevices ADQ14DC-2X digitizer with a 14 bit vertical resolution was used processing the signals.^51^ The overall time resolution of the measurement system was ≈0.230 ns and all spectra contained at least 1 × 10^7^ counts. A typical lifetime spectrum *N*(*t*) which is the absolute value of the time derivative of the positron decay spectrum, is described by

5 $$N\left(t\right)=R\left(t\right)\sum_{i=1}^{k+1}\frac{I_{i}}{\tau_{i}}e^{-t/\tau_{i}}+Background$$

where *k* is the number of different defect types contributing to the positron trapping, which are related to *k*+*1* components in the spectra with individual lifetimes τ_*i*_ and intensities *I*_*i*_ with ∑*I*_*i*_ = 1. The instrument resolution function *R*(*t*) is a sum of two Gaussian functions with distinct intensities and a relative shift both depending on the positron implantation energy, *E*_p_. *R*(*t*) was determined by measuring a reference sample, i.e. yttria-stabilized zirconia, which exhibits a known single lifetime component of 182 ± 3 ps. The background was negligible, hence assumed as zero in data analysis. All spectra were deconvoluted using a nonlinear least-squares fitting method, minimized by the Levenberg–Marquardt algorithm in the software package PALSfit,^52^ into 2 major lifetime components, which directly evidence localized annihilation at 2 different defect types (sizes; τ_1_ and τ_2_). The mean positron implantation depth ⟨*z*⟩ can be approximated by the simple material density-dependent (ρ) Makhovian function^53^

6 $$\left\langle z\right\rangle \left[nm\right]=\frac{36}{\rho }E_{p}^{1.62}\left[keV\right]$$

### Doppler Broadening Variable Energy Positron Annihilation Spectroscopy (DB-VEPAS)

DB-VEPAS measurements have been conducted at the slow positron beamline (SPONSOR).^54^ Positrons have been accelerated and monoenergetically implanted into samples in the range between *E*_p_ = 0.05–35 keV, which realizes depth profiling. Implanted positrons lose their kinetic energy due to thermalization and after short diffusion annihilate in delocalized lattice sites or localize in vacancy like defects and their agglomerations emitting at least two anticollinear 511 keV gamma photons after encountering electrons. Since at the annihilation site thermalized positrons have a small momentum compared to electrons a broadening of the 511 keV line is observed mostly due to momentum of electrons. The emitted radiation is measured by high-purity Ge detectors (energy resolution of 1.1 ± 0.1 keV). This broadening is characterized by two distinct parameters *S* and *W* defined as a fraction of the annihilation line in the middle (511 ± 1 keV) and outer regions (504–509 keV and 513–518 keV), respectively. The total area below the curve, which is utilized for normalization of both parameters, is 511 ± 16 keV. The *S*-parameter is a fraction of positrons annihilating with low momentum valence electrons and represents vacancy type defects and their concentration. The *W*-parameter approximates overlap of positron wave function with high momentum core electrons. Plotting calculated *S* as a function of positron implantation energy, *S*(*E*_p_), provides depth dependent information, whereas *S*–*W* plots are used to examine atomic surrounding of the defect site and its size (type).^55^

## Supplementary Material



## Notes

θ/2θ X-ray diffraction patterns for structural characterization; VSM measurements of gated films grown without applied magnetic field; Low temperature exchange bias in a gated CoO film; Cross-sectional TEM characterization of gated films; Average positron lifetime as a function of depth for as-grown and gated thick films; DB-VEPAS characterization of as-grown and gated films; Micromagnetic simulations of the hysteresis loops of gated films; Magnetic anisotropy of gated films by FMR and angular dependence VSM; VSM measurements of as-grown and gated thick films; VSM measurements of as-grown and gated thin films (additional measurements) (PDF)
