# Supplementary material for: Voltage-Driven Generation of Ferromagnetism in a Magneto-Ionically Active Antiferromagnet Enabling Room-Temperature Exchange Bias
Source: ACS Nano. 2026 Mar 23;20(13):10484–97. doi: 10.1021/acsnano.5c19864 (PMC13063812; doi:10.1021/acsnano.5c19864)
Supplement: Supplementary file 1 [file nn5c19864_si_001.pdf]

Supplementary Information

Voltage-driven generation of ferromagnetism in a magneto-ionically active antiferromagnet enabling room-temperature exchange bias

Simone Privitera[†,‡], Zheng Ma[†,*], Hugo Gómez-Torres[†,‡], Aitor Arredondo-López[†], Maciej Oskar Liedke[₴], Eric Hirschmann[₴], Andreas Wagner[₴], Huan Tan[†], Pau Solsona[†], Alberto Quintana[†,‡], Thiago Dias[‖], Diane Gouéré[¶], Elmer Monteblanco[¶], Dafiné Ravelosona[¶,⊥], Nuria Del-Valle[†], Carles Navau[†], Aitor Lopeandia[†,‡,*], Jordi Sort[†,‡,#,*], and Enric Menéndez[†,*]

[†]Departament de Física, Universitat Autònoma de Barcelona, 08193 Cerdanyola del Vallès, Spain.

[‡]Catalan Institute of Nanoscience and Nanotechnology (ICN2), CSIC and BIST, Campus UAB, 08193 Cerdanyola del Vallès, Spain.

[₴]Institute of Radiation Physics, Helmholtz-Zentrum Dresden–Rossendorf, Dresden 01328, Germany.

[‖]Universidade Tecnológica Federal do Paraná, Campus Dois Vizinhos, Estrada para Boa Esperança, km 04, 85660-000, Dois Vizinhos PR, Brazil.

[¶]Spin-Ion Technologies, 10 Boulevard Thomas Gobert, Palaiseau 91120, France.

[⊥]Centre de Nanosciences et de Nanotechnologies, CNRS, Université Paris-Saclay, 10 Boulevard Thomas Gobert, Palaiseau 91120, France.

[#]Institució Catalana de Recerca i Estudis Avançats (ICREA), Pg. Lluís Companys 23, 08010 Barcelona, Spain.

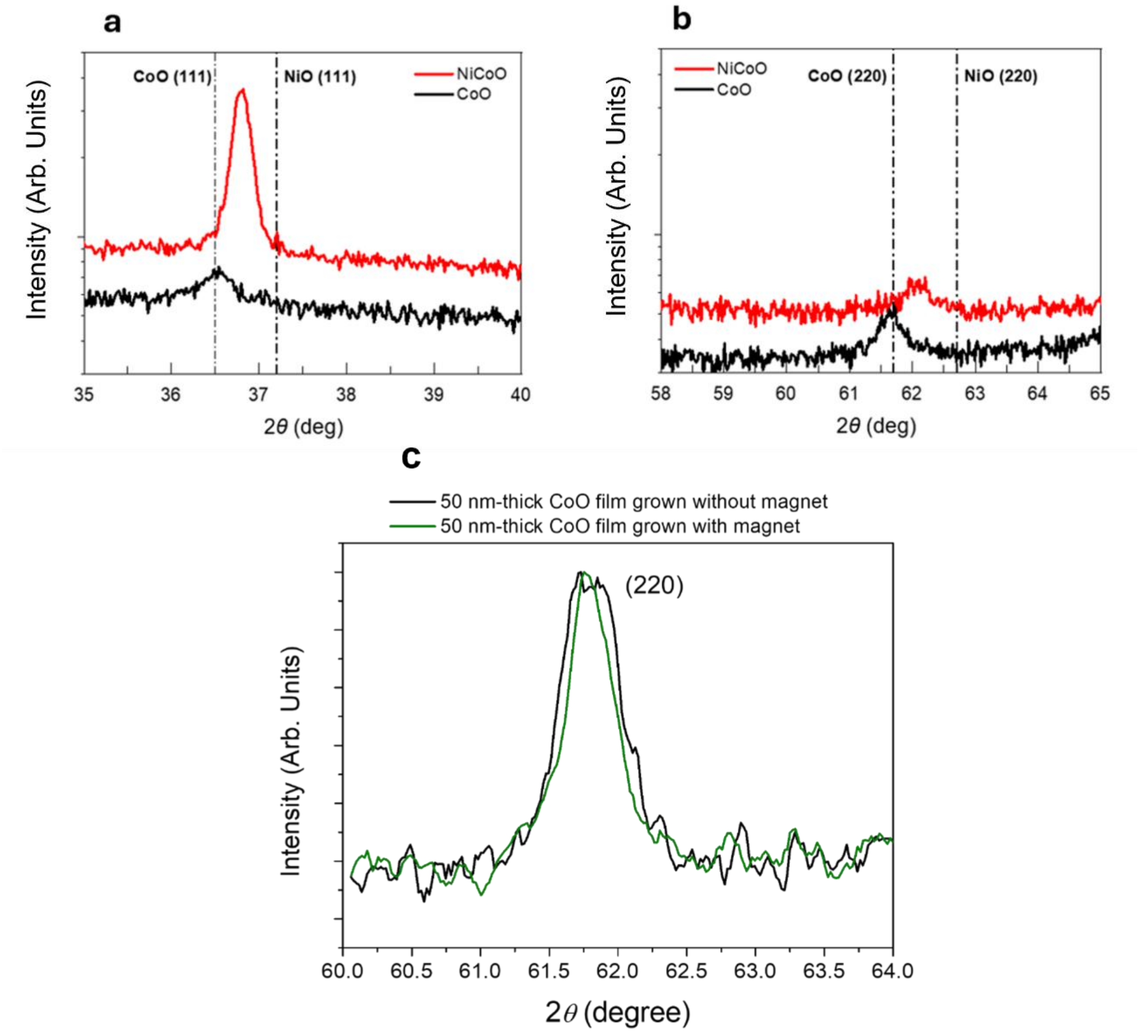

Figure S1. *θ*/2*θ* X-ray diffraction patterns of the as-grown CoO and $Ni_{0.25}Co_{0.75}O$ films. **(a)** and **(b)** 35 - 45 and 58 - 65 *θ*/2*θ* ranges: the XRD peaks of $Ni_{0.25}Co_{0.75}O$ fall between (111) and (220) planes of rock-salt CoO and NiO, respectively, indicating the formation of rock-salt-type $Ni_{0.25}Co_{0.75}O$. **(c)** *θ*/2*θ* XRD patterns of 200 nm-thick CoO films grown with and without magnet (recorded in the 60-64° range). The pattens have been smoothed for clarity.

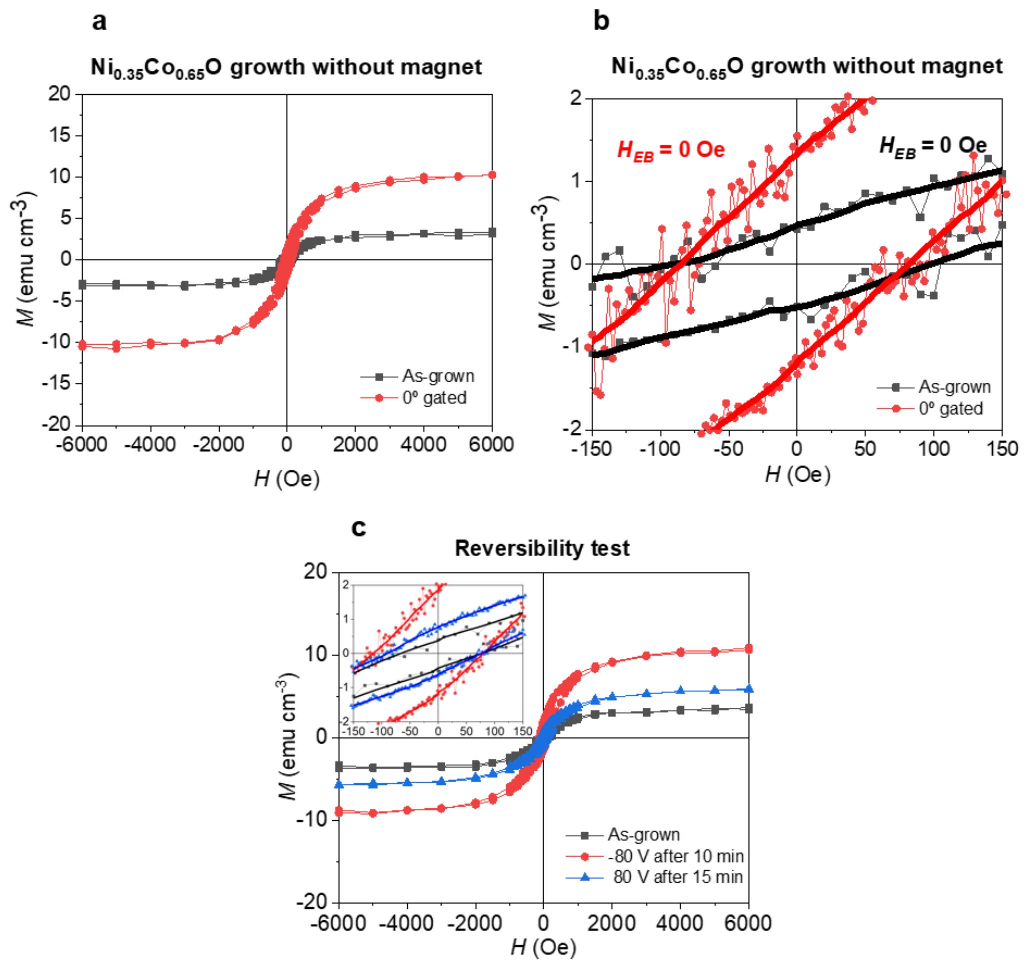

Figure S2. (a) Hysteresis loops of as-grown and gated (-80V for 10 min) $Ni_{0.35}Co_{0.65}O$ films grown without applied magnetic field. **(b)** Magnified view of the central region of the hysteresis loops in panel (a), showing that the loops exhibit no shift along the applied magnetic field axis. **(c)** Hysteresis loops corresponding to as-grown, gated (-80V for 10 min), and recovered (*i.e.*, -80V for 10 min & 80V for 15 min) $Ni_{0.35}Co_{0.65}O$ films grown under applied magnetic field.

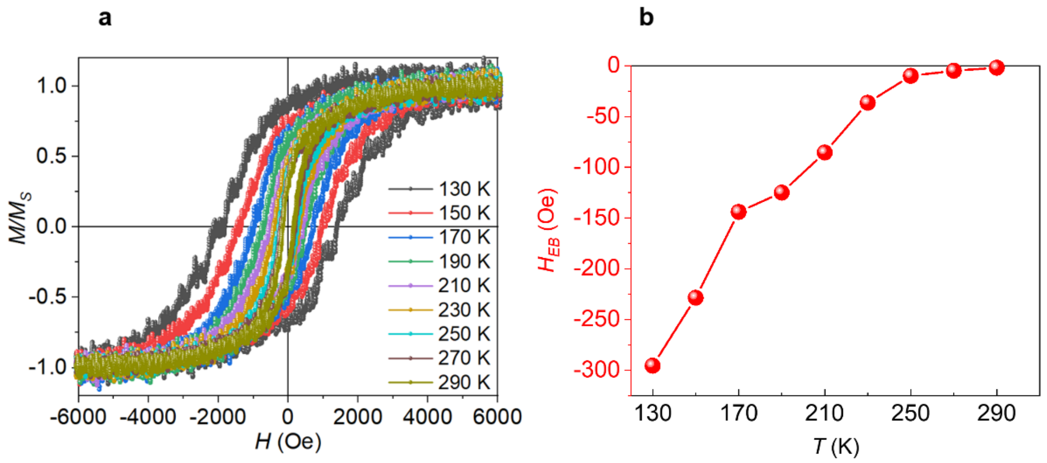

Figure S3. Low temperature exchange bias in a gated CoO film. **(a)** Normalized magnetization (M/M_S) *vs*. applied magnetic field *(H)* measurements along the field-cooling direction for a 50 nm-thick CoO film gated at −80 V for 10 min. The sample was field-cooled from room temperature down to 130 K under 10 kOe, and *M-H* loops were recorded upon heating in 20 K steps up to 290 K. **(b)** The exchange bias shift (H_{EB}) is plotted as a function of temperature *(T)*.

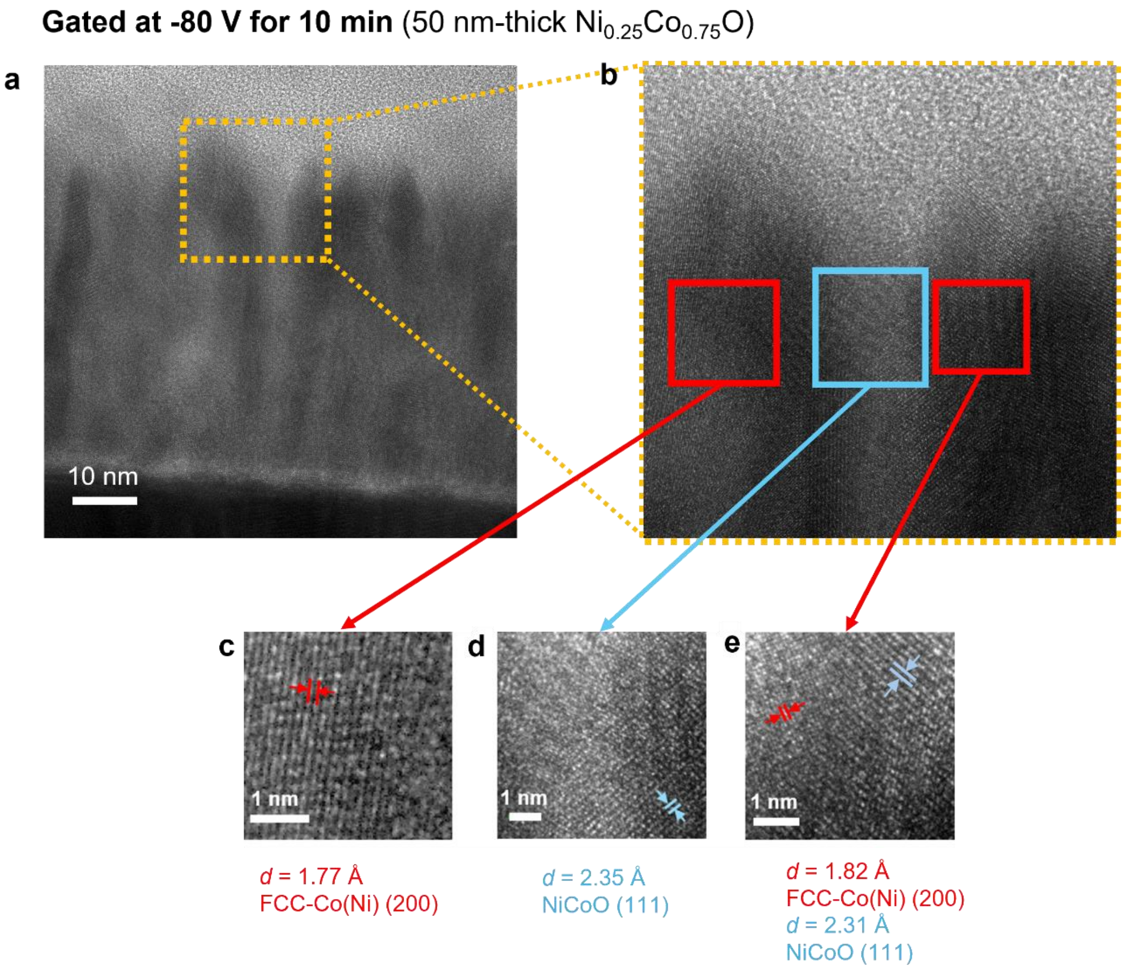

Figure S4. Cross-sectional TEM characterization of a $Ni_{0.25}Co_{0.75}O$ film gated at -80V for 10 min. (a) TEM image highlighting a grain boundary between columnar grains. **(b)** Zoom-in of panel (a). **(c-e)** High-resolution TEM images of regions surrounding the grain boundary (c and e) and inside the grain boundary (d).

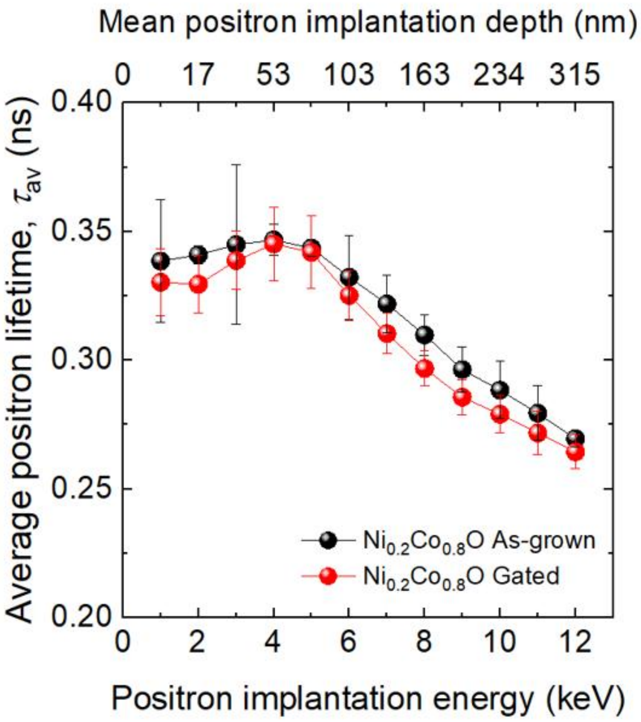

Figure S5. Average positron lifetime as a function of positron implantation energy (*i.e.*, film depth) for an as-grown and a gated (-80V for 2 h) 200 nm-thick $Ni_{0.2}Co_{0.8}O$ films.

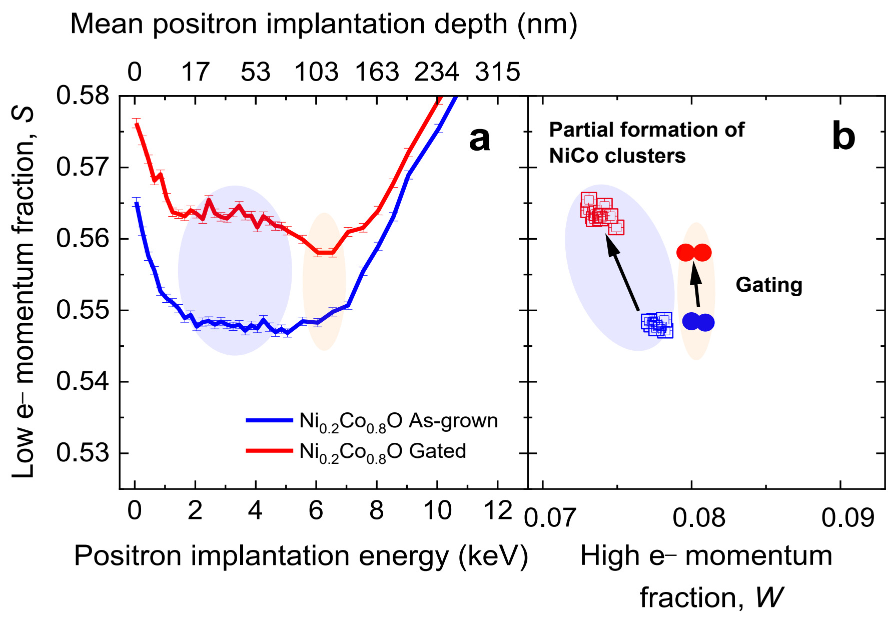

Figure S6. Structural defect characterization by DB-VEPAS. (a) *S*-parameter as a function of positron implantation energy and mean positron implantation depth for 200 nm-thick $Ni_{0.2}Co_{0.8}$ films in as-grown and gated (-80V for 2 h) states. **(b)** *S*-*W* relation plotted for data points from the top part (in a purplish contour) and bottom region (in a soft orange contour), close to the film/buffer interface.

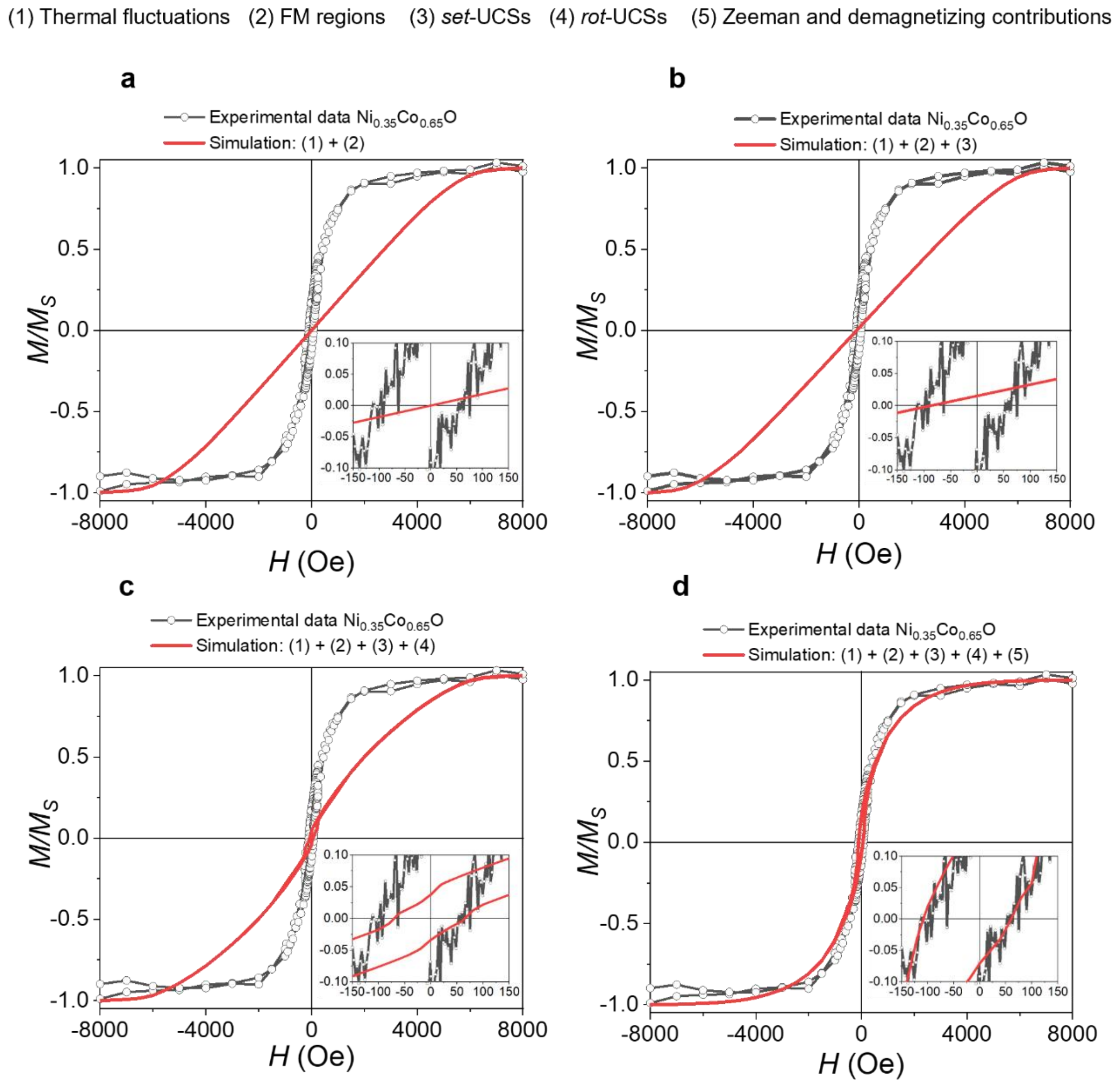

Figure S7. Micromagnetic simulations of the hysteresis loop of a $Ni_{0.35}Co_{0.65}O$ film actuated with -80 V for 10 min. (a) Thermal fluctuations and NiCo grains are considered only. **(b)** *Set*-UCS are further included on top of considerations in panel (a). **(c)** *Rot*-UCS are considered on top of considerations in panel (b). **(d)** Zeeman effects and demagnetizing field are taken into account on top of considerations in panel (c).

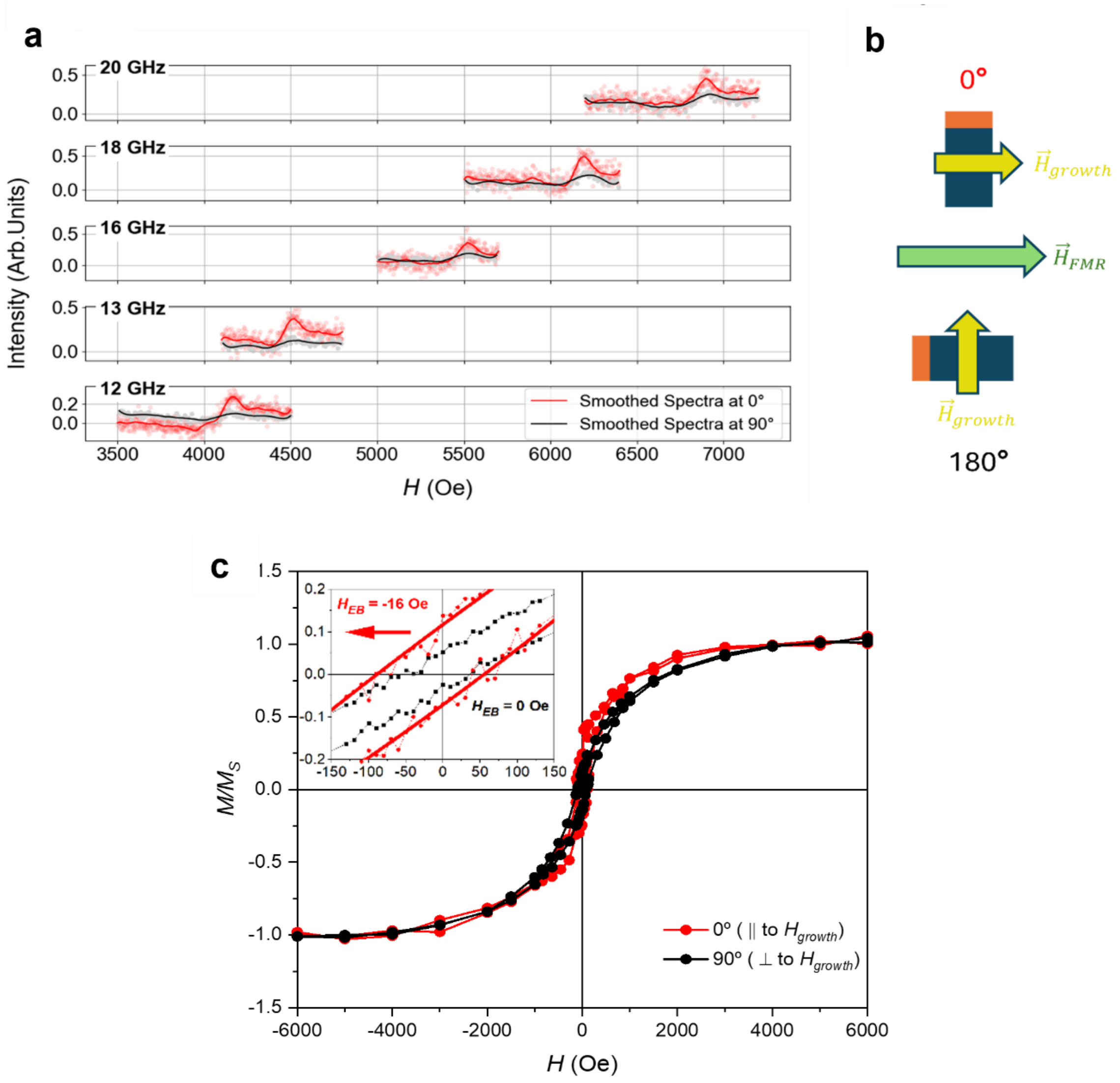

Figure S8. In-plane magnetic anisotropy analysis of a $Ni_{0.25}Co_{0.75}O$ film actuated with -80 V for 10 min. (a) Broadband FMR measurements at multiple frequencies. **(b)** Schematic of the sample orientation showing the in-plane magnetic field H_{FMR} applied parallel (0º) and perpendicular (90º) to H_{growth}. **(c)** *M-H* measurements of a $Ni_{0.35}Co_{0.65}O$ film subjected to −80 V for 10 min recorded along (0º) and across (90º) the direction the magnetic field used during growth. An inset showing a zoom-in of the coercive-field region is included.

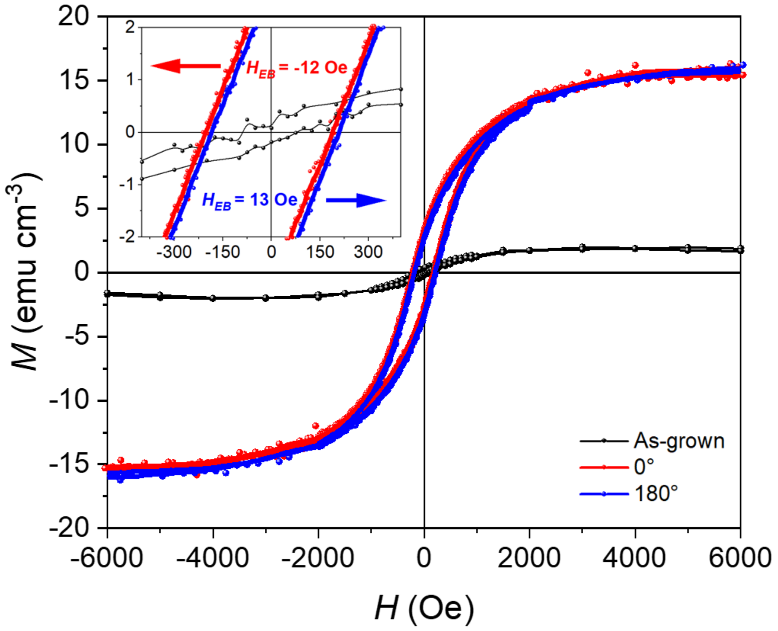

Figure S9. *M-H* measurements of a 200 nm-thick $Ni_{0.2}Co_{0.8}O$ film in as-grown state and after being gated at -80 V for 2 h measured parallel (0º) and antiparallel (180º) to the applied magnetic field used during growth, H_{growth}. The inset shows a zoom-in of the measurements, highlighting the coercive-field region.

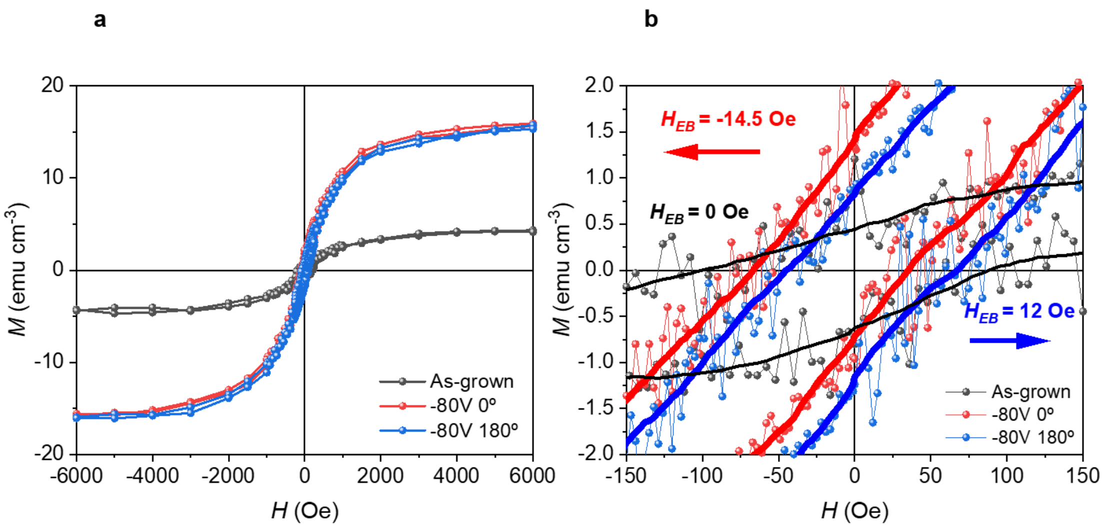

Figure S10. (a) *M-H* measurements of a 50 nm-thick $Ni_{0.25}Co_{0.75}O$ film in as-grown state and after being gated at -80 V for 10 min measured parallel (0º) and antiparallel (180º) to the applied magnetic field used during growth, H_{growth}. **(b)** Zoom-in of panel (a), highlighting the coercive-field region.

Table S1. Interplanar spacings *d* obtained from the FFT analysis shown in Figure 3b,c, corresponding to an as-grown $Ni_{0.25}Co_{0.75}O$ film.

# Ring/Spot	Experimental *d* (Å)	Reference *d* (Å)	Plane	Phase, PDF card (Relative intensity)
1	2.48	2.45	(111)	CoO, PDF 00-001-1227 (67%)
2	2.12	2.12	(200)	CoO, PDF 00-001-1227 (100%)
3	1.49	1.50	(220)	CoO, PDF 00-001-1227 (100%)
4	1.24	1.23	(222)	CoO, PDF 00-001-1227 (40%)

Table S2. Interplanar spacings *d* obtained from the FFT analysis shown in Figure 3b,c, corresponding to a $Ni_{0.25}Co_{0.75}O$ film gated at -80 V for 10 min. FCC stands for face-centered cubic and HCP for hexagonal close-packed.

# Ring/Spot	Experimental *d* (Å)	Reference *d* (Å)	Plane	Phase, PDF card (Relative intensity)
1	2.48	2.45	111	CoO, PDF® 00-001-1227 (67%)
2	2.12	2.12	200	CoO, PDF® 00-001-1227 (100%)
3	1.73	1.77	200	FCC-Co, PDF® 00-015-0806 (40%)
4	1.88	1.90	101	HCP-Co, PDF® 00-005-0727 (100%)
